# Ubiquitination-dependent regulation of ferroptosis in ischemic heart and brain

**DOI:** 10.1016/j.redox.2026.104249

**Published:** 2026-06-08

**Authors:** Yi-Yue Zhang, Xing-Yu Long, Jing Tian, Xiu-Ju Luo, Jun Peng

**Affiliations:** a Department of Pharmacy, Union Hospital, Tongji Medical College, Huazhong University of Science and Technology, Wuhan, 430022, China; b Department of Pharmacology, Xiangya School of Pharmaceutical Sciences, Central, South University, Changsha, 410013, China; c Department of Laboratory Medicine, The Third Xiangya Hospital of Central South University, Changsha, 410013, China; d Department of Clinical Pharmacy, Hunan University of Medicine General Hospital, Huaihua, 418000, China

**Keywords:** Ubiquitination, Ferroptosis, Ischemia/reperfusion, Myocardial infarction, Ischemic stroke

## Abstract

Ischemia-reperfusion (I/R) injury is a major cause of tissue damage after myocardial infarction and ischemic stroke. Ferroptosis is an iron-dependent form of regulated cell death (RCD) marked by phospholipid peroxidation, and it is an important contributor to I/R-related injury. Although oxidative stress and lipid peroxidation are common features of I/R injury, they do not fully explain why ferroptosis sensitivity increases during reperfusion. Recent studies have shown that ferroptosis is also influenced by the ubiquitin system. Changes in ubiquitination and deubiquitination regulate key proteins involved in iron metabolism, lipid remodeling, and antioxidant defense, thereby altering cell susceptibility to ferroptosis under I/R stress. This review summarizes current evidence showing how ubiquitin-dependent regulation controls ferroptosis in both cardiac and cerebral I/R injury. We focus on mechanisms that disrupt iron homeostasis, weaken antioxidant defenses, increase oxidation-sensitive membrane lipids, and alter organelle stress responses. We also highlight the shared mechanisms in the heart and brain, while noting that the main ubiquitin-regulated control points differ between these tissues. In addition, we discuss emerging therapeutic strategies targeting selected E3 ubiquitin ligases and deubiquitinating enzymes. A better understanding of the ubiquitin-ferroptosis axis may support the development of more precise therapies for ischemic injury in the cardiovascular and cerebrovascular systems.

## Highlights

• Ubiquitination regulates ferroptosis in cardiac and cerebral I/R injury.
• The key ferroptosis checkpoints differ between the heart and brain.
• E3 ligases and deubiquitinating enzymes are potential targets for anti-ferroptosis therapy.

## Graphical abstract

Overview of this article. Ubiquitination regulates ferroptosis in ischemic heart and brain injury via affecting iron balance, antioxidant defense, phospholipid peroxidation driven by polyunsaturated fatty acids, and organelle stress responses. Although these processes all lead to membrane damage and ferroptosis in both heart and brain, the underlying disease context is different in the heart and brain because they have different metabolic needs, cellular sensitivity, and local responses to ischemia-reperfusion. Targeting ubiquitination-related ferroptosis pathways may therefore provide a promising treatment strategy for ischemic heart disease and stroke.

**Figure:**
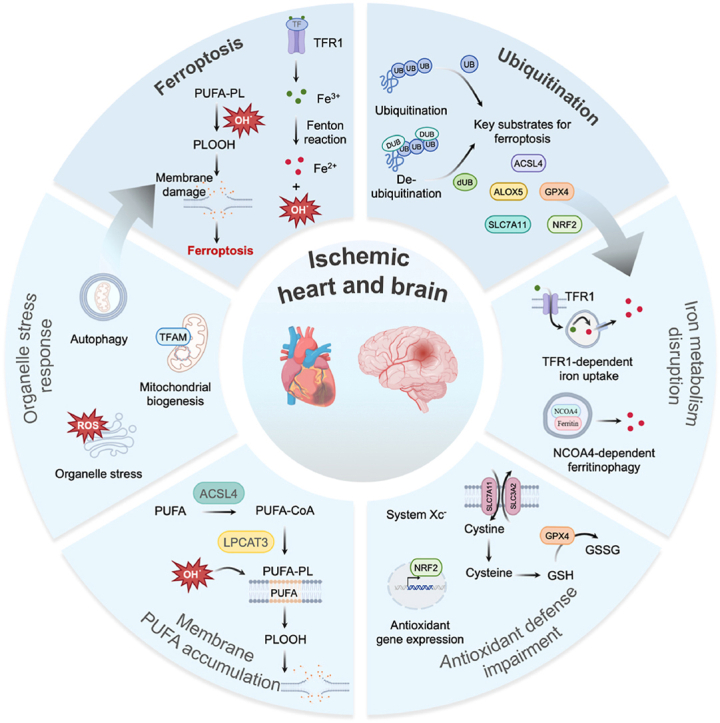

## 1 Introduction

Ischemic heart disease and ischemic stroke remain leading causes of death and long-term disability worldwide [1]. Timely reperfusion is essential for limiting tissue loss, but restoration of blood flow often causes ischemia/reperfusion (I/R) injury and adds further cellular damage [2,3]. This limits functional recovery and remains a major challenge in clinical treatment. I/R injury results from combined metabolic disturbance, calcium overload, excessive production of reactive oxygen species (ROS), and inflammatory activation [[4], [5], [6]]. Together, these changes trigger multiple forms of regulated cell death (RCD) in ischemic tissues [[7], [8], [9]].

In this network of cell death pathways, ferroptosis, an iron-dependent form of regulated cell death marked by uncontrolled lipid peroxidation [10], contributes greatly to irreversible cell loss during I/R [[11], [12], [13]]. It is closely associated with intracellular iron accumulation, glutathione (GSH) depletion, and loss of glutathione peroxidase 4 (GPX4) activity. These features overlap with the metabolic and redox changes caused by ischemic stress and help explain why I/R creates conditions that favor ferroptosis [14]. However, oxidative stress and lipid peroxidation alone do not fully explain why ferroptosis sensitivity increases during reperfusion. The key factors that determine cellular vulnerability to ferroptosis under I/R conditions are still not fully clear.

Under acute ischemic stress, cell fate is significantly influenced by post-translational control, which allows rapid and reversible changes in protein function beyond transcriptional regulation [15]. Among these pathways, the ubiquitin system plays a central role. E1 activating enzymes, E2 conjugating enzymes, and E3 ubiquitin ligases attach ubiquitin to target proteins, thereby regulating the stability, localization, and signaling activity of the target proteins [16]. Ubiquitination was first linked mainly to proteasomal degradation, but it is now known to regulate many cellular processes. This broader role is further shaped by different ubiquitin chain types and by deubiquitinating enzymes (DUBs), which remove ubiquitin from target proteins [17]. Because ischemic injury develops rapidly, this system is well-suited for controlling cell fate through rapid and reversible changes in key proteins. In this context, ubiquitin-dependent regulation is not only part of the overall stress response but also a direct mechanism influencing early ferroptosis sensitivity during I/R. Recent studies support this view. In myocardial I/R injury, the E3 ubiquitin ligase Parkin promotes ubiquitination and degradation of acyl-CoA synthetase long-chain family member 4 (ACSL4), which reduces lipid peroxidation and improves cardiomyocyte survival [18]. In cerebral I/R injury, the deubiquitinating enzyme ubiquitin-specific protease 14 (USP14) stabilizes GPX4 by removing ubiquitin chains, strengthens antioxidant defense, and limits ferroptosis [19]. These findings show that ubiquitination acts directly on core ferroptosis-related proteins rather than serving only as a secondary response to cell death.

This review summarizes current evidence that supports ubiquitination as an important regulator of ferroptosis during I/R. We focus on four interconnected areas, including iron handling, lipid remodeling and lipid peroxidation, antioxidant defense, and organelle quality control. We also compare the common mechanisms and tissue-specific differences between the heart and brain. On this basis, we clarify how ubiquitin-dependent regulation shapes ferroptosis during I/R stress and to discuss emerging therapeutic strategies that selectively target disease-related ubiquitin pathways in ischemic cardiovascular and cerebrovascular injury.

## 2 Ferroptosis in cardiac and cerebral I/R injury

### 2.1 Core mechanisms of ferroptosis

Ferroptosis is a form of RCD driven by iron-dependent lipid peroxidation, with particular vulnerability in membrane phospholipids enriched in polyunsaturated fatty acids (PUFAs) [20]. It develops through synergistic changes in iron metabolism, membrane lipid composition, and antioxidant defense. These changes collectively lead to excessive accumulation of lipid hydroperoxides, disrupting membrane integrity, and ultimately resulting in irreversible cell death [10]. Disruption of iron homeostasis is an early and critical event in ferroptosis. Iron enters cells predominantly via transferrin receptor 1 (TFR1)-mediated endocytosis [21]. After internalization, transferrin-bound ferric iron (Fe^3+^) is released in endosomal compartments and reduced to ferrous iron (Fe^2+^) by six-transmembrane epithelial antigen of the prostate 3 (STEAP3), enabling iron to enter the cytosolic labile iron pool [22]. Under normal conditions, excess iron is stored in ferritin, which limits its redox activity. During ferroptosis, ferritin undergoes selective degradation through nuclear receptor coactivator 4 (NCOA4)-dependent ferritinophagy, releasing redox-active Fe^2+^ into the cytosolic labile iron pool [23]. At the same time, impaired iron export caused by ferroportin (FPN) dysfunction further increases intracellular iron retention [24]. These changes expand the pool of redox-active iron and promote Fenton reaction-driven lipid oxidation.

Excess redox-active iron promotes ferroptosis by catalyzing radical formation and driving chain-propagating lipid peroxidation in PUFA-rich membrane phospholipids. ACSL4 activates PUFAs such as arachidonic acid (AA) and adrenic acid (AdA), and lysophosphatidylcholine acyltransferase 3 (LPCAT3) incorporates them into membrane phospholipids, particularly phosphatidylethanolamines (PE), thereby enriching membranes with highly oxidizable PUFA-PE species [18,25,26]. When the labile iron pool expands, Fe^2+^-dependent reactions initiate lipid radical formation and rapidly amplify peroxidation across the membrane, a process that can be further accelerated by lipoxygenases [27]. The resulting accumulation of phospholipid hydroperoxides disrupts membrane organization and integrity, ultimately triggering ferroptotic membrane failure and cell death.

**Fig. 1:**
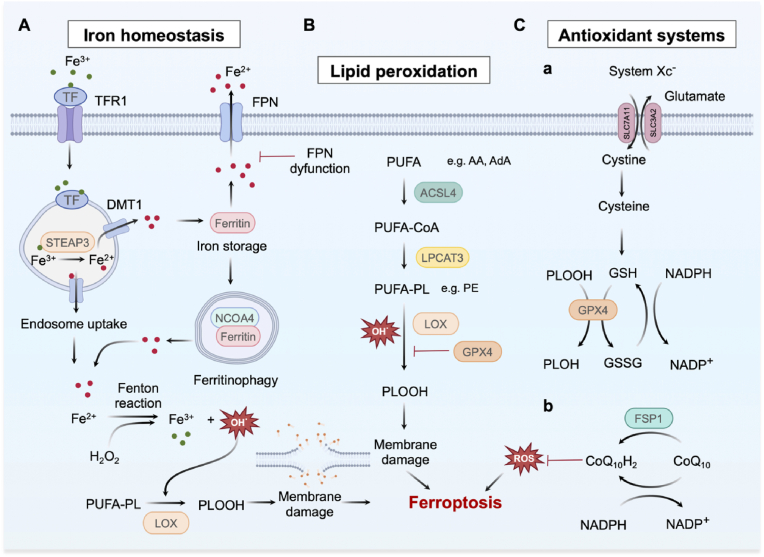
**Core mechanisms of ferroptosis.** (A) Iron homeostasis. TF-bound Fe^3+^ enters cells through TFR1-mediated endocytosis. In endosomes, Fe^3+^ is reduced to Fe^2+^ by STEAP3, and Fe^2+^ is then transported into the cytosol by DMT1. Excess intracellular iron is stored in ferritin, whereas iron export depends on FPN. Dysfunction of FPN promotes intracellular iron retention. Ferritinophagy, mediated by NCOA4, releases ferritin-bound iron and expands the labile iron pool. Redox-active Fe^2+^ reacts with H_2_O_2_ through the Fenton reaction to generate OH·, which initiates oxidative damage and promotes ferroptosis. (B) Lipid peroxidation. PUFAs, including AA and AdA, are activated by ACSL4 to form PUFA-CoA and are then incorporated into phospholipids by LPCAT3, generating peroxidation-sensitive PUFA-PLs, such as PE-containing species. LOX catalyzes the oxidation of PUFA-PLs to PLOOH. Excessive accumulation of PLOOH disrupts membrane integrity, leading to membrane damage and ferroptosis. (C) Antioxidant defense systems. (a) The system Xc^−^/GSH/GPX4 pathway is a major defense against ferroptosis. System Xc^−^, composed of SLC7A11 and SLC3A2, imports cystine in exchange for glutamate. Intracellular cystine is reduced to cysteine and used for GSH synthesis. GPX4 uses GSH to reduce PLOOH to PLOH, thereby limiting lipid peroxidation. (b) The FSP1-CoQ_10_ pathway acts independently of GPX4. FSP1 uses NADPH to reduce CoQ_10_ to CoQ_10_H_2_, which functions as a lipophilic radical-trapping antioxidant and suppresses ROS-driven lipid peroxidation. Failure of these antioxidant systems promotes ROS accumulation, membrane damage, and ferroptosis. Abbreviations: TF, Transferrin; TFR1, transferrin receptor 1; STEAP3, six-transmembrane epithelial antigen of the prostate 3; DMT1, divalent metal transporter 1; FPN, ferroportin; NCOA4, nuclear receptor coactivator 4; H_2_O_2_, hydrogen peroxide; OH·, hydroxyl radicals; PUFA-PL, phospholipids; LOX, lipoxygenases; PUFA, polyunsaturated fatty acid; PLOOH, phospholipid hydroperoxide; ACSL4, acyl-CoA synthetase long-chain family member 4; LPCAT3, lysophosphatidylcholine acyltransferase 3; GPX4, glutathione peroxidase 4; SLC7A11, solute carrier family 7 member 11; SLC3A2, solute carrier family 3 member 2; GSH, glutathione (reduced); GSSG, glutathione (oxidized); PLOH, phospholipid alcohol; CoQ_10_, ubiquinone; CoQ_10_H_2_, ubiquinol; NADPH, nicotinamide adenine dinucleotide phosphate (reduced); NADP^+^, nicotinamide adenine dinucleotide phosphate (oxidized).

Protection against lipid peroxidation depends on antioxidant defense systems. Among them, the system xc^−^/GSH/GPX4 axis is the main protective pathway. System xc^−^ imports cystine into cells, and this function depends largely on solute carrier family 7 member 11 (SLC7A11), a core transporter that helps maintain intracellular cysteine and GSH availability [28]. Cystine is then reduced to cysteine and used for GSH synthesis. GPX4 uses GSH to reduce phospholipid hydroperoxides to non-toxic lipid alcohols [29]. Loss of GPX4 activity, depletion of intracellular GSH, or reduced cystine availability rapidly leads to lipid peroxide accumulation and ferroptosis. Ferroptosis suppressor protein 1 (FSP1) provides an additional defense pathway that acts independently of GPX4. FSP1 localizes to cellular membranes and maintains coenzyme Q10 (CoQ10) in its reduced antioxidant form, which limits lipid radical propagation [30]. Both the GPX4-dependent and FSP1-dependent pathways require adequate nicotinamide adenine dinucleotide phosphate (NADPH), which makes ferroptosis sensitivity closely related to cellular redox status [31]. Overall, ferroptosis reflects the balance between iron-driven lipid peroxidation and the capacity of antioxidant systems to restrain oxidative membrane damage. Fig. 1 summarizes the core molecular events underlying ferroptosis.

### 2.2 How I/R triggers ferroptosis: similarities and differences between heart and brain

I/R injury creates conditions that favor ferroptosis via disrupting iron homeostasis, weakening antioxidant defenses, and altering membrane lipid composition. During ischemia, adenosine triphosphate (ATP) depletion limits GSH synthesis and reduces GPX4 activity, which gradually weakens the ability of cells to clear lipid hydroperoxides [32]. Moreover, hypoxia and nutrient deprivation promote intracellular iron accumulation by increasing TFR1-dependent iron uptake and activating NCOA4-dependent ferritinophagy [33,34]. As the labile iron pool expands and antioxidant defenses decline, cells enter a state that becomes more vulnerable to ferroptosis, even before obvious ferroptosis occurs. Our previous work, together with studies from other groups, have suggested that ferroptosis remains limited during ischemia and becomes much more prominent after reperfusion [14,20]. When oxygen supply is restored, a rapid burst of ROS accelerates iron-dependent lipid peroxidation in cells that have already been primed by ischemic stress. Furthermore, calcium overload activates phospholipases and promotes the release of PUFAs from membrane phospholipids [35]. These PUFAs are then reincorporated into membrane phospholipids through ACSL4-and LPCAT3-dependent pathways, which increases the abundance of oxidation-sensitive membrane lipids. During reperfusion, the combined effects of ROS overproduction, elevated redox-active iron, and PUFA-enriched membranes create the key conditions for efficient ferroptosis initiation [14,20].

Although ferroptosis is a shared feature of both cardiac and cerebral I/R injury, the main factors that shape its sensitivity differ between these tissues. In the heart, cardiomyocytes contain abundant mitochondria, which support intense ROS generation and dynamic iron flux during I/R. Previous studies have shown that myoglobin degradation can release large amounts of heme-bound iron, while changes in iron storage and export further affect intracellular iron availability [36,37]. In addition, the strong dependence of cardiomyocytes on fatty acid oxidation helps maintain a continuous PUFA supply [20]. Together, these features place redox stress, iron handling, and lipid metabolism at the center of ferroptosis regulation during cardiac I/R. In the brain, ferroptosis is shaped more strongly by cellular heterogeneity and microenvironmental disruption. Previous studies have shown that blood-brain barrier (BBB) damage during I/R allows abnormal iron entry into the brain parenchyma and causes uneven iron distribution among neural cell populations [38,39]. Neurons and glial cells do not respond equally to this stress. Oligodendrocytes appear to be particularly vulnerable because myelin membranes are rich in both iron and PUFAs [40]. Other studies have also revealed that neurons are exposed to glutamate excitotoxicity during cerebral I/R [41]. Excess extracellular glutamate may impair the system xc^−^-GSH axis by inhibiting the cystine/glutamate antiporter system xc^−^, thereby reducing cystine uptake, limiting GSH synthesis, and weakening antioxidant defense [42]. These combined changes increase ferroptosis sensitivity in specific neural cell populations during cerebral I/R.

**Fig. 2:**
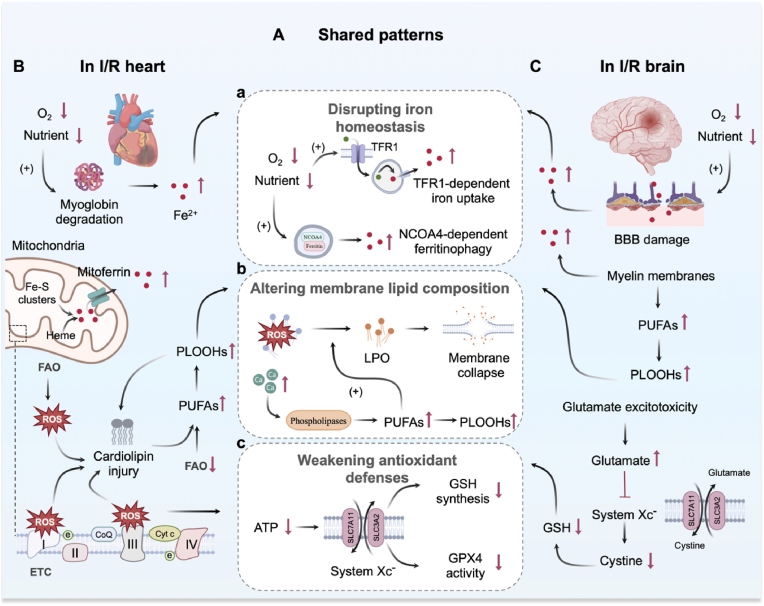
**Organ-specific features of ferroptosis in the heart and brain.** (A) Shared patterns in cardiac and cerebral I/R injury. Ischemia and nutrient deprivation create a ferroptosis-prone state through three interconnected changes. (a) Iron homeostasis is disrupted, with increased TFR1-dependent iron uptake and enhanced NCOA4-dependent ferritinophagy, leading to expansion of the labile iron pool. (b) Membrane lipid composition is altered. ROS promote lipid peroxidation, while phospholipase activation increases PUFA release, which further enhances the formation of lipid peroxides and PLOOH, ultimately resulting in membrane collapse. (c) Antioxidant defenses are weakened. ATP depletion suppresses system Xc^−^ activity, reduces cystine availability, limits GSH synthesis, and decreases GPX4 activity, thereby lowering cellular resistance to lipid peroxidation. (B) In the heart. Cardiac I/R injury is characterized by metabolic and mitochondrial involvement in ferroptosis. Under ischemic stress, myoglobin degradation releases Fe^2+^, adding to intracellular iron overload. Mitochondrial dysfunction further promotes iron accumulation through mitoferrin-dependent transport and disrupts Fe–S cluster biogenesis and heme metabolism. At the same time, ETC dysfunction causes excessive ROS production, and iron-dependent oxidative reactions aggravate cardiolipin peroxidation and mitochondrial injury. Reduced FAO during I/R is associated with PUFA accumulation, which provides substrates for lipid peroxidation and increases ferroptosis susceptibility during reperfusion. (C) In the brain. The main characteristics of cerebral I/R injury are barrier disruption and excitotoxic stress. BBB damage promotes abnormal iron entry into the brain, while myelin membrane injury increases the local availability of PUFAs and thereby favors PLOOH formation. Moreover, glutamate excitotoxicity inhibits system Xc^−^, reduces cystine import, weakens GSH synthesis, and further impairs antioxidant defense. These changes increase the sensitivity of neural cells to ferroptosis during reperfusion. Abbreviations: FAO, fatty acid oxidation; ETC, mitochondrial electron transport chain; TFR1, transferrin receptor 1; NCOA4, nuclear receptor coactivator 4; LPO, lipid peroxidation; PUFA, polyunsaturated fatty acid; PLOOH, phospholipid hydroperoxide; GSH, glutathione (reduced); GPX4, glutathione peroxidase 4; BBB, blood-brain barrier.

Overall, ferroptosis is a common pathological component of I/R injury in both the heart and brain, but the factors that drive it are not identical. In cardiomyocytes, ferroptosis is influenced more strongly by metabolic demand, redox stress, and lipid use. In the brain, it is shaped more strongly by barrier disruption, local environmental changes, and differences among neural cell types. Fig. 2 illustrates these organ-specific patterns of ferroptosis regulation following I/R.

## 3 The ubiquitin system and stress signaling

### 3.1 Ubiquitination and deubiquitination

Ubiquitination is an ATP-dependent post-translational modification that regulates protein stability, activity, and subcellular localization [43]. This process begins with ubiquitin-activating enzymes (E1), which activate ubiquitin and transfer it to ubiquitin-conjugating enzymes (E2). In human cells, this step is mediated mainly by ubiquitin-like modifier activating enzyme 1 (UBA1) and UBA6 [44,45]. Activated ubiquitin is then passed to E2 enzymes, which work with ubiquitin ligases to support substrate modification. E3 ligases are the main determinants of substrate selectivity because they recognize target proteins and catalyze ubiquitin transfer [46]. E3 ligases are generally divided into three major families. Really interesting new gene (RING)-type E3 ligases act mainly as scaffolds and promote direct transfer of ubiquitin from E2 to the substrate [47]. Homologous to the E6-associated protein C-terminus (HECT)-type E3 ligases first form a transient intermediate with ubiquitin before transferring it to the substrate [48]. RING-between-RING (RBR)-type E3 ligases combine features of both mechanisms and transfer ubiquitin through an active-site cysteine residue [49]. Through these different catalytic modes, E3 ligases control protein degradation, signaling, and intracellular trafficking.

**Fig. 3:**
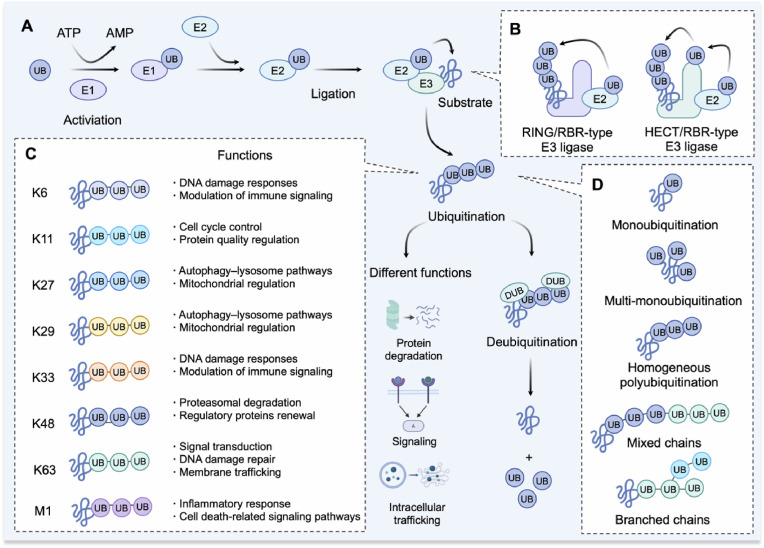
**Mechanisms and functional results of the ubiquitin system.** (A) Ubiquitination and deubiquitination. Ubiquitination proceeds through an ATP-dependent enzymatic cascade involving E1, E2, and E3 enzymes. UB is first activated by E1, transferred to E2, and then conjugated to substrate proteins through the action of E3 ligases. Depending on the ubiquitin signal, substrate modification can lead to different functional outcomes, including proteasomal degradation, signaling, and intracellular trafficking. DUBs remove ubiquitin from modified substrates, thereby reversing ubiquitination and releasing free UB for reuse. (B) Major E3 ligase families and catalytic modes. RING-type E3 ligases act mainly as scaffolds and facilitate direct transfer of UB from E2 to the substrate. HECT-type E3 ligases first form a covalent intermediate with UB before transferring it to the substrate. RBR-type E3 ligases share features of both mechanisms and also transfer UB through an intermediate step. (C) Ubiquitin chain linkages and functional consequences. Different ubiquitin linkage types encode distinct cellular functions. K6-linked chains are associated with DNA damage responses and modulation of immune signaling. K11-linked chains are linked to cell cycle control and proteostasis. K27- and K29-linked chains are involved mainly in autophagy-lysosome pathways and mitochondrial regulation. K33-linked chains are associated with DNA damage responses and immune signaling. K48-linked chains mainly target substrates for proteasomal degradation and turnover of regulatory proteins. K63-linked chains function mainly in signal transduction, DNA repair, and membrane trafficking. M1-linked chains are linked to inflammatory responses and cell death-related signaling pathways. (D) Ubiquitin chain topologies. Substrates can undergo monoubiquitination or multi-monoubiquitination, and can also be modified by polyubiquitin chains with different architectures, including homogeneous, mixed, and branched chains. These different topologies further expand the signaling and functional diversity of the ubiquitin system. Abbreviations: UB, ubiquitin; ATP, adenosine triphosphate; AMP, adenosine diphosphate; E1, ubiquitin-activating enzyme; E2, ubiquitin-conjugating enzyme; E3 ubiquitin ligase; DUB, deubiquitinating enzyme; K6/11/27/29/33/48/63, Lysine 6/11/27/29/33/48/63; M1, the N-terminal methionine. RING, really interesting new gene; HECT, homologous to the E6-associated protein C-terminus; RBR, RING-between-RING.

The reversibility of ubiquitin signaling depends on DUBs, which remove ubiquitin from target proteins and can also change the length and linkage of ubiquitin chains [50]. In this way, they alter substrate fate, prevent degradation, or preserve protein function. DUBs do more than terminate ubiquitin signals. They also shape the strength and timing of ubiquitin-dependent responses [51]. Their expression and activity are tightly regulated, often through interaction with E3 ligases or incorporation into multiprotein complexes. This shows that DUBs act as active regulators rather than simple antagonists of ubiquitination. Together, ubiquitination and deubiquitination form a dynamic regulatory cycle that allows cells to respond rapidly to stress, including ischemic injury. The ubiquitination status of a protein reflects the balance between E3 ligase-driven modification and DUB-mediated removal. Because this cycle is fast and reversible, it can alter protein behavior more quickly than transcriptional regulation while maintaining substrate specificity. The core components and regulatory logic of the ubiquitin system are summarized in Fig. 3.

### 3.2 Ubiquitin chain types and functional outcomes

The regulatory versatility of the ubiquitin system has been shown to extend beyond substrate selection and to depend largely on the structural diversity of ubiquitin chains [52]. A single ubiquitin molecule contains eight potential linkage sites for chain elongation, including seven lysine residues (K6, K11, K27, K29, K33, K48, and K63) and the N-terminal methionine (M1) [53]. Ubiquitin molecules can be connected via any of these positions, with linkage specificity determined by defined E2-E3 enzyme pairings [54]. As a consequence, polyubiquitin chains vary in length, linkage composition, and branching architecture, giving rise to distinct three-dimensional conformations. These structural features have been recognized as carriers of encoded information that directs how ubiquitinated proteins are interpreted and processed in the cell [52].

Interpretation of ubiquitin signals is mediated by proteins containing ubiquitin-binding domains, which preferentially recognize chain topology rather than individual ubiquitin moieties [55]. This recognition pattern allows compact and extended chains to function as distinct signals even when conjugated to the same substrate. Depending on chain architecture, ubiquitination can route proteins toward proteasomal degradation, modulate enzymatic activity, regulate subcellular localization, or promote assembly of signaling complexes [56]. In this manner, variation in ubiquitin chain structure is translated into specific biological outcomes, enabling precise control over protein fate and signaling behavior.

Among the different linkage types, K48-linked polyubiquitin chains have been firmly established as the canonical signal for proteasomal degradation [57]. These chains adopt a compact conformation that has been efficiently recognized by receptors of the 26S proteasome, thereby supporting substrate engagement, unfolding, and subsequent degradation [58]. This pathway plays a central role in proteostasis and in the turnover of key regulatory proteins, particularly under conditions of cell stress. K63-linked ubiquitin chains, in contrast, form more extended structures and generally do not target substrates for degradation. Instead, they have been shown to act as organizational scaffolds that recruit signaling proteins and stabilize multiprotein complexes, leading to prominent roles in signal transduction, DNA damage repair, and membrane trafficking [[59], [60], [61]]. Linear ubiquitin chains assembled via the N-terminal methionine provide an additional regulatory layer. Generated by the linear ubiquitin chain assembly complex (LUBAC), these chains reinforce PPIs within signaling platforms and have been reported to amplify inflammatory and cell death-related signaling pathways [62,63].

Beyond these well-characterized linkages, additional ubiquitin chain types contribute to more specialized regulatory functions. K11-linked chains are closely associated with cell cycle control and have been reported to participate in endoplasmic reticulum-associated protein quality regulation [64,65]. K27- and K29-linked chains are related to autophagy-lysosome pathways and mitochondrial regulation [66,67], whereas K6- and K33-linked chains have been found to implicate in DNA damage responses and modulation of immune signaling [[68], [69], [70]]. Importantly, ubiquitin chains formed in vivo are often heterogeneous. Mixed or branched chains containing multiple linkage types can be assembled on a single substrate, allowing sequential or combinatorial instructions such as transient signaling followed by timed proteasomal degradation [71]. These complex architectures expand the functional repertoire of ubiquitin signaling while increasing regulatory precision. The core features of ubiquitin chain signaling are summarized in Fig. 3.

### 3.3 Ubiquitin signaling during ischemic stress

The ubiquitin-proteasome system is a rapid stress-responsive pathway that links ischemic injury to changes in ferroptosis-related proteins. This function is closely related to the biochemical requirement of ubiquitin conjugation. Because activation of ubiquitin by E1 enzymes depends on ATP, ubiquitination is highly sensitive to changes in cellular energy status [72]. During ischemia, ATP depletion suppresses ubiquitin conjugation on a broad scale. After reperfusion, restored energy supply reactivates ubiquitin-dependent protein turnover. These changes occur before most transcriptional responses and directly affect the stability of proteins that control redox balance and lipid peroxidation.

Oxidative stress further reshapes ubiquitin signaling during I/R. ROS can modify redox-sensitive components of the ubiquitin machinery and shift the balance between ubiquitination and deubiquitination [73,74]. This change affects important regulators of antioxidant defense, including proteins involved in cystine transport and lipid peroxide detoxification. As a result, oxidative stress can rapidly change the abundance of proteins that suppress ferroptosis and reduce cellular resistance to lipid peroxidation. During reperfusion, inflammatory signals further strengthen this process. Damage-associated molecular patterns (DAMPs) released from injured cells activate pattern-recognition receptors and trigger ubiquitin-dependent signaling pathways [75]. This may create conditions that favor the induction or activation of specific E3 ligases and deubiquitinating enzymes involved in the regulation of ferroptosis-related proteins. Current evidence further suggests that this selective ubiquitin-dependent remodeling is an important determinant of ferroptosis sensitivity during ischemic stress. Stress-responsive E3 ligases and deubiquitinating enzymes continuously adjust the stability of central ferroptosis regulators, including SLC7A11 and GPX4 [76,77]. Even slight changes in the ubiquitination status of these proteins can strongly affect intracellular glutathione availability and the ability of cells to clear lipid peroxides. Furthermore, ubiquitin-dependent regulation of iron-related and lipid metabolic proteins further changes susceptibility to ferroptosis under ischemic conditions [78,79]. In this way, the combined effects of metabolic stress, oxidative injury, and inflammation allow the ubiquitin system to convert I/R stress into changes in ferroptosis sensitivity.

## 4 Ubiquitin-dependent control of ferroptosis in cardiac and cerebral I/R injury

Ubiquitin-dependent regulation influences ferroptosis during cardiac and cerebral I/R injury through both direct and indirect mechanisms. Direct regulation refers to E3 ligases or DUBs acting on ferroptosis-related proteins, such as ACSL4, GPX4, SLC7A11, NCOA4, iron regulatory protein 2 (IRP2), or TfR1, thereby changing their ubiquitination, stability, or activity. Indirect regulation refers to ubiquitin-dependent control of upstream transcription factors, signaling proteins, or stress-responsive regulators that subsequently alter ferroptosis effectors. Thus, the term “ubiquitin-dependent regulation” in this review includes both direct post-translational control of ferroptosis proteins and upstream ubiquitin-regulated signaling events. The strength of evidence varies among studies. Strong causal evidence usually requires substrate interaction, target ubiquitination, protein stability changes, ferroptosis-related functional readouts, and rescue experiments. Findings based mainly on changes in expression or ubiquitination levels are interpreted more cautiously as supportive or correlative evidence. The following sections discuss these effects in four interrelated aspects, including iron metabolism, lipid remodeling, antioxidant defense, and organelle quality control.

The timing of ubiquitin-dependent events is also important. Early reperfusion is marked by oxygen restoration, ATP recovery, ROS burst, calcium overload, and rapid protein turnover, whereas later stages involve stronger inflammatory signaling, transcriptional remodeling, autophagy, mitochondrial quality control, and tissue repair. However, the exact timing of many ubiquitination events remains unclear because most studies rely on endpoint measurements rather than dynamic ubiquitination profiling. Representative events are summarized by their reported or likely temporal phase in Table 1.

**Table 1:** Proposed time windows of representative ubiquitin-dependent events involved in ferroptosis during cardiac and cerebral I/R injury.

Time window	Main biological features	Representative ubiquitin-dependent events	Main ferroptosis-related effect	Evidence note
Early reperfusion, minutes to hours	ATP recovery, ROS burst, calcium overload, and rapid protein turnover	USP7-p53-TfR1; HOIL-1-IRP2-TfR1/FPN1; USP14-GPX4; TRIM62-GPX4; RNF126-SLC7A11	Rapid regulation of iron uptake, iron export, GPX4 stability, cystine uptake, and lipid peroxide detoxification	These events are likely to affect early ferroptosis sensitivity, but exact timing requires further validation
Intermediate reperfusion, hours	Propagation of lipid peroxidation, activation of stress signaling, and remodeling of lipid metabolism	Parkin-ACSL4; RNF5-ACSL4; NEDD4L-ACSL4; FBXO10-ACSL4; TRIM67-ACSL4; MDM2-ACSL4; TRIM65-ALOX5	Control of peroxidizable phospholipid formation and enzymatic lipid peroxidation	Functional roles have been reported, but time-resolved ubiquitination assays remain limited
Late reperfusion and remodeling, hours to days	Inflammation, autophagy, mitophagy, mitochondrial renewal, chromatin regulation, and tissue remodeling	USP19-Beclin1; PINK1/Parkin-mediated mitophagy; RNF34-PGC1α; TRIM13-Nur77; PRC1-H2AK119ub-HSP27; USP46-SNX5	Regulation of organelle quality control, mitochondrial ROS, autophagy-related ferroptosis, and stress adaptation	These pathways are often related to sustained injury or repair responses, although overlap with early phases is possible

Abbreviations: I/R, ischemia-reperfusion; ATP, adenosine triphosphate; ROS, reactive oxygen species; USP7, ubiquitin-specific protease 7; TfR1, transferrin receptor 1; IRP2, iron regulatory protein 2; HOIL-1, heme-oxidized IRP2 ubiquitin ligase 1; USP14, ubiquitin-specific protease 14; FPN1, ferroportin 1; GPX4, glutathione peroxidase 4; TRIM62, tripartite motif containing protein 62; RNF126, ring finger protein 126; SLC7A11, solute carrier family 7 member 11; ACSL4, acyl-CoA synthetase long-chain family member 4; RNF5, ring finger protein 5; NEDD4L, neural precursor cell expressed developmentally down-regulated 4-like; FBXO10, F-box only protein 10; TRIM67, tripartite motif containing protein 67; MDM2, murine double minute 2; TRIM65, tripartite motif containing protein 65; ALOX5, arachidonate lipoxygenase 5; USP19, ubiquitin-specific protease 19; PINK1, phosphatase and tensin homolog (PTEN)-induced kinase 1; RNF34, ring finger protein 34; PGC1α, peroxisome proliferator activated receptor gamma coactivator 1 alpha; TRIM13, tripartite motif containing protein 13; Nur77, nuclear receptor subfamily 4 group A member 1; PRC1, polycomb repressive complex 1; H2AK119ub, histone H2A Lys119 ubiquitination; HSP27, heat shock protein 27; USP46, ubiquitin-specific protease 46; SNX5, sorting nexin 5.

### 4.1 Iron metabolism

Disordered iron homeostasis is a key cause of ferroptosis in cardiac and cerebral I/R injury. Recent studies have shown that the ubiquitin system regulates this process at several levels, including iron uptake, intracellular iron storage, mitochondrial iron handling, and ferritin-dependent iron release. Ubiquitination alters the unstable iron pool and affects the sensitivity of ischemic tissues to ferroptosis via changing the stability of iron-related proteins or their upstream regulators.

In myocardial I/R injury, there is an important link between deubiquitination and iron accumulation. Our laboratory have found that USP7 stabilizes p53 by blocking its ubiquitin-dependent degradation [9]. The increase in p53 then upregulates TfR1, enhances transferrin dependent iron uptake, and promotes intracellular iron overload during reperfusion. Through this pathway, USP7 expands the redox active iron pool and increases ferroptotic death in cardiomyocytes. *Xu* et al. have further revealed that USP7 also stabilizes phosphatase and tensin homolog (PTEN) and worsens ferroptosis by inhibiting phosphatidylinositol 3-kinase (PI3K)/protein kinase B (AKT) signaling [80]. These findings suggest that USP7 promotes myocardial ferroptosis through both iron accumulation and stress related signaling. Ferritin-derived iron release is another major source of iron overload in the ischemic heart. *Xue* et al. have demonstrated that OTU domain-containing ubiquitin aldehyde-binding protein 1 (OTUB1) directly deubiquitinates NCOA4 and maintains its stability [81]. NCOA4 is the ferritin cargo receptor required for ferritinophagy. Therefore, OTUB1-driven NCOA4 stabilization maintains ferritin turnover, promotes the release of ferritin-bound iron into unstable iron pools, and enhances ferroptosis during myocardial I/R.

Another iron related mechanism in myocardial I/R involves the processing of mitochondrial iron. *Zhang* et al. have reported that frataxin limits ferroptotic injury by maintaining myocardial iron homeostasis, while NHL repeat containing E3 ubiquitin protein ligase 1 (NHLRC1) acts as the upstream E3 ligase that targets frataxin for degradation [82]. Loss of frataxin weakens mitochondrial iron sequestration, increases redox active iron, and makes cardiomyocytes more vulnerable to ferroptosis during reperfusion. This discovery extends the role of ubiquitination beyond iron absorption and points out that mitochondrial iron buffering is another important step in cardiac ferroptosis.

In cerebral I/R injury, ubiquitin-dependent iron metabolism control is more directly linked to the classical iron regulation pathway. Our lab has shown that heme-oxidized IRP2 ubiquitin ligase 1 (HOIL-1) is an important upstream regulator of IRP2 in ischemic stroke [78]. Reduced HOIL-1 weakens the ubiquitin dependent turnover of IRP2 and causes IRP2 accumulation. Increased IRP2 then raises TfR1 expression and suppresses FPN1, which enhances iron uptake and reduces iron export at the same time. Through this pathway, neurons tend to retain iron, resulting in intracellular iron overload, and are more prone to ferroptosis after reperfusion. Direct regulation of iron import has also been reported in cerebral I/R. *Su* et al. have shown that neural precursor cell expressed developmentally down-regulated 4-like (NEDD4L) suppresses ferroptosis by promoting the ubiquitin-dependent downregulation of TfR1 [83]. This can reduce transferrin-mediated iron uptake and limit neuronal iron accumulation.

Ferritinophagy-dependent iron release is another major source of ubiquitin-controlled ferroptotic iron in the reperfused brain. *Li* et al. have reported that USP14 directly deubiquitinates and stabilizes NCOA4, which enhances ferritinophagy and promotes the release of ferritin bound iron into the labile iron pool [33]. Through this pathway, ferritin stores are converted into redox active iron and neuronal ferroptosis is amplified during cerebral I/R. In contrast, *Zhang* et al. have identified a protective ubiquitin-dependent pathway that acts on the same ferritinophagy machinery at an upstream level [84]. They have shown that USP18 stabilizes fat mass and obesity-associated protein (FTO), and maintained FTO activity suppresses NCOA4 expression after transcription. As a result, ferritinophagy-dependent iron release is reduced, ferritin-derived iron mobilization is limited, and neuronal ferroptosis is attenuated in cerebral I/R. Thus, both USP14 and USP18 affect NCOA4 dependent ferritinophagy, but they act in opposite directions. USP14 promotes ferroptosis by directly stabilizing NCOA4, whereas USP18 suppresses ferroptosis by reducing NCOA4 expression through FTO. These studies indicate that the ubiquitin system is not simply associated with iron overload during reperfusion. It directly controls how iron is taken up, retained, buffered, and released, and in this way influences ferroptosis in the ischemic heart and brain.

### 4.2 Lipid remodeling and lipid peroxidation

Lipid remodeling is another key factor that determines ferroptosis in cardiac and cerebral I/R injury. Iron overload provides the basis for oxidative damage, but the degree of membrane injury depends largely on whether polyunsaturated fatty acids are incorporated into phospholipids that are easy to oxidize. Recent studies have shown that the ubiquitin system mainly regulates this process by controlling the stability of lipid remodeling enzymes, especially ACSL4. In this way, the ubiquitin system changes the content of easily oxidized membrane lipids, thereby affecting ferroptosis during reperfusion.

In myocardial I/R injury, several ubiquitin-dependent pathways act on ACSL4. *Xiao* et al. have reported that Parkin, an RBR type E3 ligase, promotes the ubiquitin-dependent degradation of ACSL4, which reduces lipid peroxidation and suppresses ferroptosis in cardiomyocytes [18]. This finding shows that Parkin limits ferroptotic membrane changes in the ischemic heart by reducing ACSL4 dependent peroxidizable phospholipids. A similar protective effect has been reported for ring finger protein 5 (RNF5). *Shuai* et al. have shown that RNF5 directly increases ACSL4 ubiquitination and accelerates its degradation during myocardial I/R, which lowers lipid peroxidation and reduces ferroptotic injury [85]. NEDD4L is another E3 ligase that acts on the same target in the heart. *Qiu* et al. have found that neural precursor cell expressed developmentally down regulated 4 like, or NEDD4L, promotes ACSL4 ubiquitination and degradation and thereby restrains cardiomyocyte ferroptosis during myocardial reperfusion [79]. In this pathway, yes associated protein (YAP) does not directly ubiquitinate ACSL4. Instead, it transcriptionally increases NEDD4L expression, which then promotes ACSL4 ubiquitination and degradation. Thus, this pathway represents upstream transcriptional regulation that converges on ubiquitin-dependent control of ACSL4.

In addition to ACSL4, other enzymes related to lipid peroxidation are also regulated by ubiquitination in myocardial I/R. *Deng* et al. have demonstrated that tripartite motif containing protein 65 (TRIM65) promotes the ubiquitination of arachidonate lipoxygenase 5 (ALOX5), reduces its protein stability, and thereby limits lipid peroxide formation and ferroptosis [86]. Unlike ACSL4 related regulation, which mainly affects phospholipid remodeling and substrate supply, the TRIM65 and ALOX5 pathway more directly limits the enzymatic spread of lipid peroxidation. This suggests that ubiquitination can suppress ferroptosis at different steps of lipid damage.

Cerebral I/R shows a similar ubiquitin-dependent pattern, and ACSL4 remains the main target. *Sun* et al. have reported that cytochrome P450 family 1 subfamily B member 1 (CYP1B1)-derived 20-hydroxyeicosatetraenoic acid (20-HETE) activates protein kinase C (PKC) signaling to induce the E3 ligase F-box only protein 10 (FBXO10), which promotes ubiquitination and degradation of ACSL4 [87]. Reduced ACSL4 limits the formation of peroxidation-prone phospholipids, thereby decreasing lipid peroxidation and neuronal ferroptosis after reperfusion. Other studies have identified additional E3 ligases that also target ACSL4 in the ischemic brain. *Xiao* et al. have shown that TRIM67 suppresses cerebral ferroptosis by promoting ACSL4 ubiquitination and reducing ACSL4 protein levels, which lowers lipid peroxidation during cerebral I/R injury [88]. Similarly, *Jin* et al. have found that RNF146 also targets ACSL4 for ubiquitin-dependent downregulation, which further supports the central role of ACSL4 in ischemic neural tissue [89]. *Ji* et al. have extended this finding by showing that murine double minute 2 (MDM2) also suppresses ferroptosis in cerebral I/R through regulation of ACSL4 [90]. These studies indicate that multiple E3 ligases in the brain, similar to those in the heart, act on ACSL4 to control membrane lipid composition and ferroptosis sensitivity. The ubiquitin system does not act directly on oxidized lipids. Instead, it influences how easily ischemic cells form and maintain membrane lipids that are prone to peroxidation.

### 4.3 Antioxidant defense

Failure of antioxidant defense is a critical step in ferroptosis during cardiac and cerebral I/R. Iron overload and lipid remodeling create the conditions for phospholipid oxidation, but ferroptosis progresses only when cells can no longer clear lipid peroxides. Studies show that the ubiquitin system regulates antioxidant defense at multiple levels. It can directly modify SLC7A11 and GPX4, or indirectly affect upstream regulators such as p53, Kelch-like ECH-associated protein 1 (KEAP1), nuclear factor erythroid 2-related factor 2 (NRF2)-related signaling, and other stress-responsive proteins. Thus, antioxidant-related pathways include both direct post-translational regulation of ferroptosis effectors and indirect regulation through transcriptional or signaling programs.

In myocardial I/R injury, ubiquitin-dependent suppression of the cystine-GSH-GPX4 pathway is closely linked to p53 signaling. As discussed in Section 4.1, p53 can also increase ferroptosis sensitivity by enhancing TfR1-dependent iron uptake through the USP7-p53-TfR1 pathway. Here, p53 acts through a different but complementary mechanism. *Gong* et al. have reported that USP38 stabilizes p53 by reducing its ubiquitin-dependent degradation [91]. The accumulated p53 then represses SLC7A11 expression, reduces cystine uptake, weakens glutathione synthesis, and promotes ferroptosis during reperfusion. Our work has identified a related but different pathway associated with cylindromatosis (CYLD). We have found that spermatogenesis associated protein 2 (SPATA2), enhances CYLD-mediated deubiquitination of p53 and prevents its degradation, which leads to p53 accumulation [92]. The stabilized p53 then suppresses SLC7A11, impairs cystine import, reduces GSH availability, and weakens GPX4 -dependent removal of lipid peroxides. Thus, although USP38 and CYLD act through different deubiquitinating pathways, they both converge at the same p53 and SLC7A11 checkpoints and promote myocardial ferroptosis. Direct ubiquitin-dependent regulation of SLC7A11 has also been reported in ischemic injury. *Zhuang* et al. have shown that RNF126, an E3 ligase, promotes the ubiquitin-dependent degradation of SLC7A11, which impairs cystine uptake and weakens intracellular antioxidant defense [93]. Unlike the p53-related pathways, RNF126 acts directly on SLC7A11 and provides a more direct route for the loss of the cystine GSH defense program.

In addition to cystine uptake, antioxidant defense in myocardial I/R is also regulated at the transcriptional level. *Yin* et al. have reported that HECT domain and ankyrin repeat containing E3 ubiquitin protein ligase 1 (HACE1) suppresses ferroptosis by maintaining NRF2 signaling and preserving the expression of downstream antioxidant genes involved in redox balance [94]. Since NRF2 supports multiple anti-ferroptosis pathways, including SLC7A11 and GPX4-related defenses, HACE1-mediated NRF2 regulation represents a broader ubiquitin-dependent mechanism that strengthens antioxidant capacity before lipid peroxide clearance fails. *Wang* et al. have demonstrated that TRIM11 promotes ubiquitination and degradation of nucleosome assembly protein 1-like 1 (NAP1L1), thereby reactivating transcription of antioxidant genes, reducing ROS accumulation and lipid peroxidation, and ultimately suppressing cardiomyocyte ferroptosis during myocardial I/R [95].

Compared with the heart, cerebral I/R shows more direct ubiquitin-dependent regulation of the terminal antioxidant machinery. A representative example is GPX4, the key phospholipid hydroperoxide reductase that directly prevents ferroptotic membrane damage. *Huang* et al. have demonstrated that TRIM62 promotes K48-linked ubiquitination of GPX4 and accelerates its proteasomal degradation, which weakens phospholipid peroxide clearance and aggravates neuronal ferroptosis after ischemic reperfusion [96]. Similarly, *Zhao* et al. have reported that kelch like protein 8 (KLHL8) also targets GPX4 for ubiquitin-dependent downregulation [77]. This further supports GPX4 destabilization as an important mechanism that promotes ferroptosis in the ischemic brain. In contrast, deubiquitination can preserve GPX4 and restore antioxidant defense. *Zhao* et al. have shown that USP14 directly deubiquitinates GPX4 and stabilizes its protein level, thereby maintaining phospholipid peroxide detoxification and suppressing neuronal ferroptosis during cerebral I/R [19]. By acting on GPX4, ubiquitin signaling reaches the most direct enzymatic step of ferroptosis and determines whether ischemic neurons can still neutralize lipid hydroperoxides. Another important antioxidant regulator in cerebral I/R is SLC7A11. *Liu* et al. have found that USP10 deubiquitinates and stabilizes SLC7A11, thereby preserving cystine uptake and maintaining the glutathione-dependent antioxidant system in reperfused neurons [76]. This pathway complements the direct stabilization of GPX4 via maintaining the substrate supply required for GPX4 activity and plays an upstream role in inhibiting brain ferroptosis.

In addition to the cystine-GSH-GPX4 pathway, FSP1 represents another important antioxidant system that suppresses ferroptosis through the CoQ10-dependent lipid radical-trapping pathway. However, compared with GPX4 and SLC7A11, ubiquitin-dependent regulation of FSP1 has been much less studied in cardiac and cerebral I/R injury. Recent studies in other disease settings have suggested that FSP1 can be regulated by ubiquitination. For example, TRIM21-mediated K63-linked ubiquitination promotes FSP1 plasma membrane localization and strengthens ferroptosis resistance [97], while RNF126 has also been reported to regulate FSP1 ubiquitination and subcellular localization [98]. Nevertheless, direct evidence identifying specific E3 ligases or DUBs that control FSP1 in myocardial or cerebral I/R injury is still lacking. Therefore, whether FSP1 ubiquitination contributes to ferroptosis sensitivity during reperfusion remains an important question for future research.

**Fig. 4:**
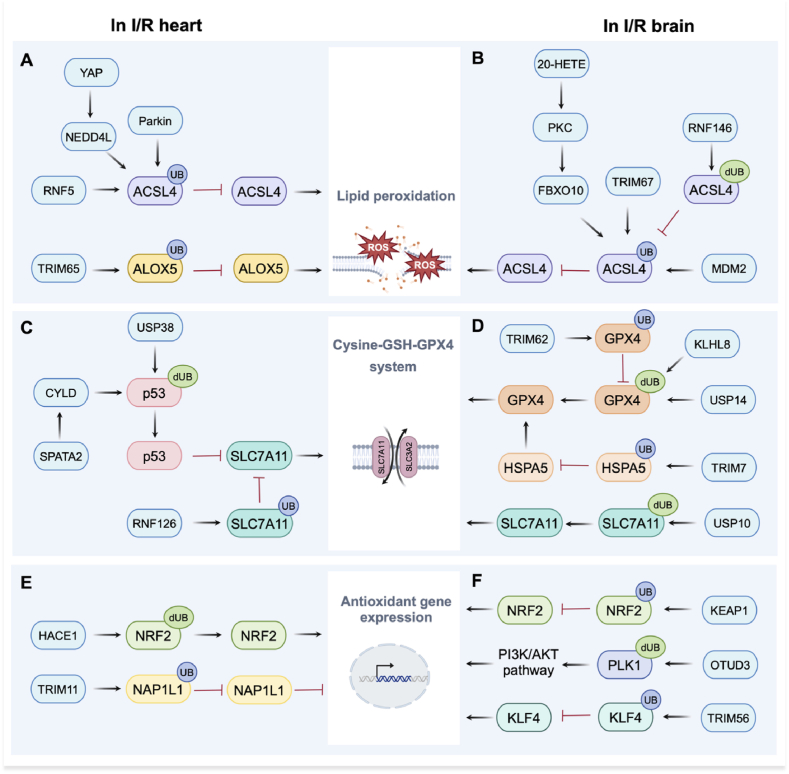
**Ubiquitin-dependent regulation of ferroptosis during myocardial and cerebral I/R injury.** (A) Lipid peroxidation in the I/R heart. During myocardial I/R, YAP promotes NEDD4L expression, and NEDD4L then enhances ACSL4 ubiquitination and downregulation, thereby limiting lipid peroxidation. Parkin and RNF5 also promote ACSL4 ubiquitination and suppress ACSL4-dependent ferroptosis. In addition, TRIM65 promotes ALOX5 ubiquitination and downregulation, which reduces lipid peroxide formation and attenuates ferroptosis. (B) Lipid peroxidation in the I/R brain. During cerebral I/R, 20-HETE activates PKC and induces FBXO10, which promotes ACSL4 ubiquitination and downregulation. TRIM67 and MDM2 also enhance ACSL4 ubiquitination, thereby suppressing ACSL4-dependent lipid peroxidation and ferroptosis. RNF146 likewise promotes ACSL4 downregulation and inhibits ferroptosis in the I/R brain. (C) The cystine-GSH-GPX4 system in the I/R heart. During myocardial I/R, USP38 deubiquitinates and stabilizes p53, whereas SPATA2 enhances CYLD-mediated deubiquitination of p53, leading to p53 accumulation. Increased p53 suppresses SLC7A11 and weakens the cystine-GSH-GPX4 defense pathway. Furthermore, RNF126 promotes SLC7A11 ubiquitination and downregulation, further impairing antioxidant defense and enhancing ferroptosis. (D) The cystine-GSH-GPX4 system and FSP1 antioxidant system in the I/R brain. During cerebral I/R, TRIM62 and KLHL8 promote GPX4 ubiquitination and downregulation, whereas USP14 deubiquitinates and stabilizes GPX4. TRIM7 promotes HSPA5 ubiquitination and downregulation, thereby weakening HSPA5-mediated protection of GPX4. USP10 deubiquitinates and stabilizes SLC7A11, preserving system Xc^−^ activity and suppressing ferroptosis. (E) Antioxidant gene expression in the I/R heart. During myocardial I/R, HACE1 promotes NRF2 stabilization and supports antioxidant gene expression. TRIM11 promotes NAP1L1 ubiquitination and downregulation, thereby relieving repression of antioxidant gene transcription and suppressing ferroptosis. (F) Antioxidant gene expression in the I/R brain. During cerebral I/R, KEAP1 promotes NRF2 ubiquitination and downregulation, leading to reduced antioxidant gene expression. OTUD3 deubiquitinates and stabilizes PLK1, thereby activating the PI3K/AKT pathway and suppressing ferroptosis. TRIM56 promotes KLF4 ubiquitination and downregulation, which weakens its anti-ferroptotic effect. Ubiquitination and deubiquitination are indicated by “UB” and “dUB,” respectively, and red blunt lines indicate inhibitory effects. Abbreviations: YAP, yes associated protein; NEDD4L, neural precursor cell expressed developmentally down-regulated 4-like; RNF5, ring finger protein 5; ACSL4, acyl-CoA synthetase long-chain family member 4; TRIM65, tripartite motif containing protein 65; ALOX5, arachidonate lipoxygenase 5; 20-HETE, 20-hydroxyeicosatetraenoic acid; PKC, protein kinase C; FBXO10, F-box only protein 10; TRIM67, tripartite motif containing protein 67; RNF146, ring finger protein 146; MDM2, murine double minute 2; USP38, ubiquitin-specific protease 38; SPATA2, spermatogenesis associated protein 2; CYLD, cylindromatosis; SLC7A11, solute carrier family 7 member 11; RNF126, ring finger protein 126; GSH, glutathione (reduced); GPX4, glutathione peroxidase 4; TRIM62, tripartite motif containing protein 62; KLHL8, kelch like protein 8; USP14, ubiquitin-specific protease 14; TRIM7, tripartite motif containing protein 7; HSPA5, heat shock protein family member A5; USP10, ubiquitin-specific protease 10; HACE1, E3 ubiquitin protein ligase 1; NRF2, nuclear factor erythroid 2-related factor 2; TRIM11, tripartite motif containing protein 11; NAP1L1, nucleosome assembly protein 1-like 1; KEAP1, Kelch-like ECH-associated protein 1; OTUD3, ovarian tumour proteases 3; PLK1, polo-like kinase 1; TRIM56, tripartite motif containing protein 56; KLF4, Krüppel-like factor 4.

Ubiquitin-dependent control of antioxidant gene expression is also seen in the brain. *Liu* et al. have reported that KEAP1, as part of the classic NRF2 ubiquitination machinery, promotes NRF2 degradation and suppresses antioxidant gene expression, which enhances ferroptosis during cerebral I/R [99]. In this context, KEAP1-dependent ubiquitination weakens ferroptosis resistance at a broader transcriptional level rather than through one single antioxidant protein. Additional studies suggest that antioxidant defense in cerebral I/R can also be regulated through other ubiquitin sensitive protective factors. *Lu* et al. have shown that TRIM7 directly binds to heat shock protein family member A5 (HSPA5), promoting its K48-linked polyubiquitination and proteasome degradation, thereby reducing GPX4 activity and amplifying ferroptosis after cerebral I/R [100]. *Cheng* et al. have reported that ovarian tumour proteases 3 (OTUD3) deubiquitinates polo-like kinase 1 (PLK1) via removing K48-linked ubiquitin chains, thereby stabilizing PLK1, activating PI3K/AKT signaling, and suppressing ferroptosis after cerebral I/R [101]. *Wang* et al. have indicated that TRIM56 promotes K48-linked polyubiquitination and proteasomal degradation of Krüppel-like factor 4 (KLF4), thereby enhancing neuronal ferroptosis after cerebral I/R [102]. Although these pathways are not part of the classic cystine-GSH-GPX4 pathway, they further show that ubiquitin-dependent regulation affects cerebral antioxidant defense through both core detoxifying proteins and upstream stress responsive regulators. These findings indicate that the ubiquitin system influences ferroptosis not only through iron accumulation and lipid remodeling, but also by determining whether ischemic cells can maintain the antioxidant capacity needed to remove phospholipid peroxides during reperfusion. Ubiquitin-dependent regulation of antioxidant pathways during myocardial and cerebral I/R injury is summarized in Fig. 4. Key ubiquitin ligases and deubiquitinating enzymes involved in ferroptosis-related signaling in ischemic diseases are listed in Table 2.

**Table 2:** Ubiquitin ligases and deubiquitinating enzymes regulating ferroptosis-related proteins in ischemic diseases.

Ubiquitin regulator	Type	Target protein(s)	Modification	Functional level	Effect on ferroptosis	Disease model	Refs
USP7	DUB	p53	NR	Iron	Promotes ferroptosis	Myocardial ischemia-reperfusion injury	[9]
OTUB1	DUB	NCOA4	NR	Iron	Promotes ferroptosis	Myocardial ischemia-reperfusion injury	[81]
NHLRC1	E3	Frataxin	NR	Iron	Promotes ferroptosis	Myocardial ischemia-reperfusion injury	[82]
Parkin	E3	ACSL4	NR	Lipid	Suppresses ferroptosis	Myocardial ischemia-reperfusion injury	[18]
NEDD4L	E3	ACSL4	NR	Lipid	Suppresses ferroptosis	Myocardial ischemia-reperfusion injury	[79]
TRIM65	E3	ALOX5	NR	Lipid	Suppresses ferroptosis	Myocardial ischemia-reperfusion injury	[86]
RNF5	E3	ACSL4	NR	Lipid	Suppresses ferroptosis	Myocardial ischemia-reperfusion injury	[85]
TRIM11	E3	NAP1L1	NR	Antioxidant	Suppresses ferroptosis	Myocardial ischemia-reperfusion injury	[95]
RNF146	E3	DAPK1	NR	Antioxidant	Suppresses ferroptosis	Myocardial ischemia-reperfusion injury	[103]
USP38	DUB	P53	NR	Antioxidant	Promotes ferroptosis	Myocardial ischemia-reperfusion injury	[91]
RNF126	E3	SLC7A11	NR	Antioxidant	Promotes ferroptosis	Myocardial ischemia-reperfusion injury	[93]
HACE1	E3	NRF2	NR	Antioxidant	Suppresses ferroptosis	Myocardial ischemia-reperfusion injury	[94]
USP7	DUB	PTEN	NR	Antioxidant	Promotes ferroptosis	Myocardial ischemia-reperfusion injury	[80]
USP13	DUB	ALDOA	K48	Antioxidant	Suppresses ferroptosis	Myocardial ischemia-reperfusion injury	[104]
CYLD	DUB	p53	NR	Antioxidant	Promotes ferroptosis	Myocardial ischemia-reperfusion injury	[92]
USP19	DUB	Beclin1	K11	Organelle	Promotes ferroptosis	Myocardial ischemia-reperfusion injury	[105]
Parkin	E3	OMM proteins	NR	Organelle	Suppresses ferroptosis	Myocardial ischemia-reperfusion injury	[106,107]
RNF34	E3	PGC1α	NR	Organelle	Promotes ferroptosis	Myocardial ischemia-reperfusion injury	[20]
PRC1	E3	Histone H2A	H2AK119ub	Organelle	Promotes ferroptosis	Myocardial ischemia-reperfusion injury	[108]
TRIM13	E3	Nur77	K48	Organelle	Promotes ferroptosis	Myocardial ischemia-reperfusion injury	[109]
NEDD4L	E3	TfR1	NR	Iron	Suppresses ferroptosis	Cerebral ischemia-reperfusion injury	[83]
USP18	DUB	FTO	NR	Iron	Suppresses ferroptosis	Cerebral ischemia-reperfusion injury	[84]
HOIL-1	E3	IRP2	NR	Iron	Suppresses ferroptosis	Cerebral ischemia-reperfusion injury	[78]
USP14	DUB	NCOA4	NR	Iron	Promotes ferroptosis	Cerebral ischemia-reperfusion injury	[33]
TRIM67	E3	ACSL4	NR	Lipid	Suppresses ferroptosis	Cerebral ischemia-reperfusion injury	[88]
MDM2	E3	ACSL4	NR	Lipid	Suppresses ferroptosis	Cerebral ischemia-reperfusion injury	[90]
FBXO10	E3	ACSL4	NR	Lipid	Suppresses ferroptosis	Cerebral ischemia-reperfusion injury	[87]
RNF146	E3	ACSL4	NR	Lipid	Suppresses ferroptosis	Cerebral ischemia-reperfusion injury	[89]
TRIM56	E3	KLF4	K48	Antioxidant	Promotes ferroptosis	Cerebral ischemia-reperfusion injury	[102]
KEAP1	E3	NRF2	NR	Antioxidant	Promotes ferroptosis	Cerebral ischemia-reperfusion injury	[99]
OTUD3	DUB	PLK1	K48	Antioxidant	Suppresses ferroptosis	Cerebral ischemia-reperfusion injury	[101]
Trim7	E3	HSPA5	K48	Antioxidant	Promotes ferroptosis	Cerebral ischemia-reperfusion injury	[100]
KDM2B	E3	OGT	NR	Antioxidant	Promotes ferroptosis	Cerebral ischemia-reperfusion injury	[110]
OTUD5	DUB	GPX4	NR	Antioxidant	Promotes ferroptosis	Cerebral ischemia-reperfusion injury	[111]
TRIM62	E3	GPX4	K48	Antioxidant	Promotes ferroptosis	Cerebral ischemia-reperfusion injury	[96]
USP14	DUB	GPX4	NR	Antioxidant	Suppresses ferroptosis	Cerebral ischemia-reperfusion injury	[19]
USP10	DUB	SLC7A11	NR	Antioxidant	Suppresses ferroptosis	Cerebral ischemia-reperfusion injury	[76]
KLHL8	E3	GPX4	NR	Antioxidant	Promotes ferroptosis	Cerebral ischemia-reperfusion injury	[77]
USP46	DUB	SNX5	NR	Organelle	Promotes ferroptosis	Cerebral ischemia-reperfusion injury	[112]

Abbreviations: USP7, ubiquitin-specific protease 7; DUB, deubiquitinating enzyme; NR, not reported; OTUB1, OTU domain-containing ubiquitin aldehyde-binding protein 1; NHLRC1, NHL repeat containing E3 ubiquitin protein ligase 1; E3, ubiquitin ligase; ACSL4, acyl-CoA synthetase long-chain family member 4; NEDD4L, neural precursor cell expressed developmentally down-regulated 4-like; TRIM65, tripartite motif containing protein 65; ALOX5, arachidonate lipoxygenase 5; RNF5, ring finger protein 5; TRIM11, tripartite motif containing protein 11; NAP1L1, nucleosome assembly protein 1-like 1; RNF146, ring finger protein 146; DAPK1, death-associated protein kinase 1; USP38, ubiquitin-specific protease 38; RNF126, ring finger protein 126; HACE1, HECT domain and ankyrin repeat containing E3 ubiquitin protein ligase 1; NRF2, nuclear factor erythroid 2-related factor 2; SLC7A11, solute carrier family 7 member 11; PTEN, phosphatase and tensin homolog; USP13, ubiquitin-specific protease 13; ALDOA, aldolase A; K48, Lysine 48; CYLD, cylindromatosis; USP19, ubiquitin-specific protease 19; K11, Lysine 11; OMM, outer mitochondrial membrane; RNF34, ring finger protein 34; PGC1α, peroxisome proliferator activated receptor gamma coactivator 1 alpha; PRC1, polycomb repressive complex 1; H2AK119ub, histone H2A Lys119 ubiquitination; TRIM13, tripartite motif containing protein 13; Nur77, nuclear receptor subfamily 4 group A member 1; TfR1, transferrin receptor 1; USP18, ubiquitin-specific protease 18; FTO, fat mass and obesity-associated protein; IRP2, iron regulatory protein 2; HOIL-1, heme-oxidized IRP2 ubiquitin ligase 1; USP14, ubiquitin-specific protease 14; NCOA4, nuclear receptor coactivator 4; TRIM67, tripartite motif containing protein 67; MDM2, murine double minute 2; FBXO10, F-box only protein 10; TRIM56, tripartite motif containing protein 56; KLF4, Krüppel-like factor 4; KEAP1, Kelch-like ECH-associated protein 1; OTUD3, ovarian tumour proteases 3; PLK1, polo-like kinase 1; Trim7, tripartite motif containing protein 7; HSPA5, heat shock protein family member A5; KDM2B, Lysine demethylase 2B; OGT, O-GlcNAc transferase; OTUD5, ovarian tumour proteases 5; GPX4, glutathione peroxidase 4; TRIM62, tripartite motif containing protein 62; USP10, ubiquitin-specific protease 10; KLHL8, kelch like protein 8; USP46, ubiquitin-specific protease 46; SNX5, sorting nexin 5.

### 4.4 Organelle quality control

In myocardial I/R injury, one major ubiquitin-related mechanism involves autophagy associated organelle turnover. *Shan* et al. have reported that USP19 promotes ferroptosis by acting on Beclin1 and regulating K11-linked ubiquitin signaling, which enhances autophagy-related ferroptosis [105]. Beclin1 is a key initiator of autophagic flux, so this finding links deubiquitination to excessive degradation activity and suggests that autophagy dysregulation can promote ferroptosis in the reperfused heart. Meanwhile, PTEN-induced kinase 1 (PINK1)/Parkin-dependent mitophagy represents a ubiquitin-driven mitochondrial quality control pathway. Following mitochondrial injury, PINK1 accumulates on the outer mitochondrial membrane and recruits Parkin, which ubiquitinates various outer mitochondrial membrane proteins, marking dysfunctional mitochondria for selective autophagic clearance [106,107]. By limiting the persistence of damaged mitochondria that produce ROS, this pathway helps maintain mitochondrial homeostasis and inhibits ferroptosis during myocardial I/R injury [113,114].

Mitochondrial biogenesis is another important point in this process. *Cai* et al. have shown that RNF34 targets peroxisome proliferator activated receptor gamma coactivator 1 alpha (PGC1α) and promotes its ubiquitin-dependent downregulation during myocardial I/R [20]. PGC1α is a key regulator of mitochondrial biogenesis and oxidative metabolism. Its deficiency disrupts mitochondrial homeostasis, increases oxidative stress, and promotes ferroptosis. This finding shows that ubiquitination can drive ferroptosis via impairing mitochondrial renewal. A related mechanism has also been reported for TRIM13. *Yang* et al. have demonstrated that TRIM13 promotes K48-linked ubiquitination of nuclear receptor subfamily 4 group A member 1 (Nur77), leading to its degradation and aggravating ferroptosis in post-ischemic myocardium [109]. Because Nur77 is involved in mitochondrial stress adaptation and metabolic regulation, its absence suggests that ubiquitin-dependent degradation of mitochondrial stress response regulators may increase ferroptosis vulnerability during cardiac injury.

Epigenetic regulation of organelle stress responses is also involved in cardiac ferroptosis. *Shi* et al. have reported that polycomb repressive complex 1 (PRC1) promotes myocardial ferroptosis by increasing histone H2A Lys119 ubiquitination (H2AK119ub), which suppresses heat shock protein 27 (HSP27) expression. Loss of HSP27 compromises mitochondrial protection, elevates mitochondrial ROS and lipid peroxidation during reperfusion, and thereby facilitates ferroptosis [108]. Unlike direct ubiquitination of an organelle protein, this pathway acts at the chromatin level but still leads to mitochondrial dysfunction and ferroptotic injury. This finding broadens the understanding of organelle quality control beyond direct protein turnover.

**Fig. 5:**
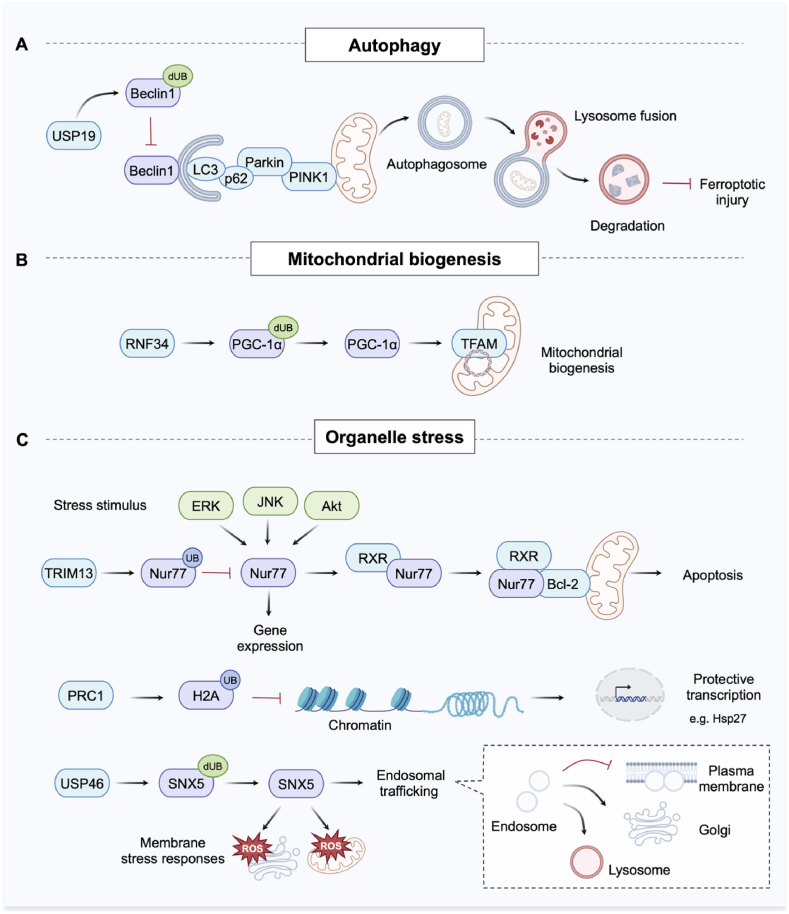
**Organelle quality control in ubiquitin-dependent regulation of ferroptosis.** (A) Autophagy. USP19 deubiquitinates and stabilizes Beclin1, thereby promoting autophagy-related ferroptosis. In parallel, the PINK1/Parkin pathway mediates mitophagy, in which damaged mitochondria are recognized, engulfed by autophagosomes, and delivered for lysosomal degradation. By controlling the turnover of injured mitochondria, this pathway regulates ferroptosis during I/R. (B) Mitochondrial biogenesis. RNF34 promotes PGC-1α downregulation, thereby limiting TFAM-associated mitochondrial biogenesis. Impaired mitochondrial renewal disrupts mitochondrial homeostasis and increases susceptibility to ferroptosis during myocardial I/R. (C) Organelle stress responses. Stress signals activate ERK, JNK, and Akt, which regulate Nur77 and promote its downstream stress-related functions. TRIM13 promotes Nur77 ubiquitination and downregulation, thereby weakening Nur77-dependent adaptive responses and aggravating ferroptosis. PRC1 promotes H2A ubiquitination, leading to repression of protective transcription, including HSP27-related stress defense at the chromatin level. USP46 deubiquitinates and stabilizes SNX5, thereby altering endosomal trafficking and membrane stress responses. Through these effects on stress adaptation, chromatin regulation, and membrane homeostasis, ubiquitin-dependent organelle control further shapes ferroptosis sensitivity during I/R. Ubiquitination and deubiquitination are indicated by “UB” and “dUB,” respectively, and red blunt lines indicate inhibitory effects. Abbreviations: USP19, ubiquitin-specific protease 19; LC3, microtubule - associated protein light chain 3; p62, Sequestosome 1; PINK1, PTEN-induced kinase 1; RNF34, ring finger protein 34; PGC1α, peroxisome proliferator activated receptor gamma coactivator 1 alpha; TFAM, mitochondrial transcription factor A; ERK, extracellular regulated protein kinase; JNK, c-Jun N-terminal kinase; Akt, protein kinase B, PKB; TRIM13, tripartite motif containing protein 13; Nur77, nuclear receptor subfamily 4 group A member 1; RXR, retinoid X receptor; Bcl-2, B-cell lymphoma 2; PRC1, polycomb repressive complex 1; H2A, histone H2A; HSP27, heat shock protein 27; USP46, ubiquitin-specific protease 46; SNX5, sorting nexin 5.

Compared to the heart, studies in cerebral I/R are still limited, but they also support the important role of ubiquitin-dependent organelle regulation. *Yang* et al. have shown that USP46 promotes ferroptosis in cerebral I/R by deubiquitinating and stabilizing sorting nexin 5 (SNX5) [112]. Because SNX5 regulates endosomal trafficking and membrane stress responses, its stabilization by deubiquitination may disrupt membrane homeostasis and increase neuronal susceptibility to ferroptosis, even without directly altering iron or lipid metabolism. The above findings indicate that the ubiquitin system influences ferroptosis not only by directly regulating iron metabolism, lipid remodeling, and antioxidant defense, but also by determining whether ischemic cells can maintain organelle integrity and adaptive capacity during reperfusion. The ubiquitin-dependent regulation of organelle quality control in ferroptosis is summarized in Fig. 5.

## 5 Therapeutic targeting of ubiquitin nodes in ferroptosis

Targeting ubiquitin-related regulators of ferroptosis is becoming a promising approach for ischemic heart and brain injury. This is because ubiquitination controls several upstream factors that affect ferroptosis during reperfusion, including iron accumulation, lipid remodeling, antioxidant defense, and organelle quality control. Instead of blocking ferroptosis only at the final stage, most current interventions act by correcting disease-related changes in ubiquitin-dependent protein stability that promote ferroptosis.

To clarify the translational relevance of these interventions, we considered their original pharmacological uses. Some agents are clinically used drugs, including Nadolol for hypertension and angina, Lurasidone for schizophrenia and bipolar depression, Dipyridamole for thromboembolic prevention, Sevoflurane for general anesthesia, and Remimazolam for procedural sedation. In contrast, compounds such as Resveratrol, Melatonin, Rosmarinic acid, and Cyanidin-3-glucoside are mainly studied as experimental or nutraceutical interventions in I/R-ferroptosis research. Therefore, these agents should be viewed as pharmacological tools or repurposing candidates rather than established anti-ferroptosis therapies for ischemic heart or brain injury.

One important therapeutic direction is the restoration of iron homeostasis. In cerebral I/R, our work have shown that Nadolol suppresses ferroptosis via targeting HOIL-1/IRP2 pathway, reversing the increase in IRP2 and TfR1 while restoring FPN1, thereby reducing neuronal iron retention and iron driven injury [78]. This finding highlights iron-related ubiquitin regulators as practical therapeutic targets in ischemic stroke.

Another major strategy is to reduce lipid peroxidation by targeting ubiquitin-dependent lipid remodeling pathways. In cerebral I/R, Tongqiao Huoxue Decoction promotes ubiquitination-dependent degradation of ACSL4 [115]. Melatonin and Remimazolam also suppress ferroptosis through MDM2-related, FBXO10-related and TRIM67-related regulation of ACSL4 [87,88,90]. In myocardial I/R, Sevoflurane suppresses ferroptosis via upregulating the E3 ligase TRIM65, which promotes ubiquitination and destabilization of ALOX5, thereby limiting lipid peroxidation-driven cardiomyocyte injury [86]. These studies suggest that regulation of ubiquitin-dependent lipid enzymes can reduce membrane vulnerability to peroxidation in both heart and brain ischemic injury.

Strengthening antioxidant defense is another major therapeutic direction. In myocardial I/R, our group have demonstrated that Lurasidone inhibits the SPATA2/CYLD deubiquitination pathway, restores p53 ubiquitination and reduces p53 accumulation, thereby relieving p53-dependent repression of SLC7A11, preserving GPX4, and suppressing ferroptosis [92]. Dipyridamole similarly preserves SLC7A11 via downregulating RNF126 [93], while Salvianolic acid B attenuates myocardial ferroptosis by reducing ubiquitin proteasome-dependent degradation of GPX4 [116]. In cerebral I/R, Piceatannol enhances USP14-dependent deubiquitination of GPX4 [19], bone marrow mesenchymal stem cell-derived exosomal USP10 stabilizes SLC7A11 [76], and Salvianolic acid B preserves GPX4 by disrupting the interaction between thrombospondin 1 (THBS1) and OTUD5 [111]. At the transcriptional level, Rosmarinic acid suppresses neuronal ferroptosis by targeting KEAP1 and NRF2-related regulation and restoring the expression of GPX4, SLC7A11, and glutamate-cysteine ligase modifier subunit (GCLM) [99]. Farudodstat reduces ferroptosis via inhibiting TRIM56 and preventing KLF4 degradation [102]. These findings show that the SLC7A11 and GPX4 and NRF2 network is the most widely used antioxidant target for therapeutic intervention.

Several studies further show that ferroptosis can be reduced by modulating ubiquitin-dependent organelle quality control. In myocardial I/R, Resveratrol and Cyanidin 3 glucoside regulate USP19-related Beclin1 signaling and suppress autophagy-related ferroptosis [105,117]. ML351 inhibits RNF34-dependent degradation of PGC1α [20], and PRT4165 suppresses PRC1-dependent H2AK119 ubiquitination [108], thereby preserving mitochondrial function and reducing ferroptosis. In addition, PINK1- and Parkin-related mitochondrial quality control has emerged as a repeated therapeutic target. Resveratrol combined with Honokiol, Trimetazidine, and Salidroside enhance protective PINK1 and Parkin signaling [[118], [119], [120]], while AP39 reduces ferroptosis by limiting excessive PINK1- and Parkin-dependent mitophagy [121].

Taken together, therapeutic targeting of ubiquitin-dependent ferroptosis in ischemic heart and brain injury can be grouped into four main directions, including correction of iron imbalance, suppression of lipid remodeling and lipid peroxide amplification, reinforcement of antioxidant defense, and preservation of organelle quality control. Among these approaches, antioxidant related interventions are currently supported by the largest number of studies, especially those acting on SLC7A11-, GPX4-, and NRF2-related pathways. Lipid-directed strategies are also prominent, with ACSL4 emerging as a repeated target, particularly in cerebral I/R. By contrast, iron-related interventions remain fewer, although they are mechanistically well supported and may be especially important in stroke.

These observations suggest that drug repurposing may be a practical route for translating ubiquitin-related anti-ferroptosis mechanisms into ischemic disease treatment. Ferroptosis-targeting interventions have shown benefits in several preclinical I/R settings, including myocardial I/R, cardiomyocyte hypoxia/reoxygenation (H/R), middle cerebral artery occlusion/reperfusion (MCAO/R), and oxygen-glucose deprivation/reoxygenation (OGD/R) models. These findings indicate that ferroptosis is modifiable during reperfusion and that ubiquitin-related nodes may serve as druggable targets. Compared with de novo drug development, repurposed drugs may offer advantages in known pharmacokinetics, safety profiles, dosing experience, and clinical accessibility.

The timing of intervention is likely critical. Early reperfusion involves ATP recovery, ROS burst, iron mobilization, and rapid lipid peroxide formation, making iron handling, ACSL4-dependent lipid remodeling, and GPX4/SLC7A11-related antioxidant defense key early targets. Later phases involve inflammation, mitochondrial dysfunction, autophagy/mitophagy, and tissue remodeling, where organelle-directed interventions may be more relevant. Since current evidence remains mainly preclinical, these effects should not be directly interpreted as established clinical benefits in myocardial infarction or ischemic stroke. Future studies should define the dosing window, tissue specificity, target engagement, delivery efficiency, and safety of these agents in disease-relevant I/R models. Representative agents and their current clinical use or development status are summarized in Table 3.

**Table 3:** Therapeutic agents targeting ubiquitin-dependent regulation of ferroptosis pathways.

Therapeutic agent	Targeted ubiquitin node	Ferroptosis pathway	Molecular effect	Disease model	Therapeutic effect	Current clinical use/status	Refs
Sevoflurane	TRIM65-ALOX5 axis	Lipid	Activates TRIM65 mediated ubiquitination of ALOX5 and decreases ALOX5 protein stability	Myocardial ischemia-reperfusion injury	Attenuates ferroptosis, inflammation apoptosis, and myocardial injury	Clinically used inhalational anesthetic for induction and maintenance of general anesthesia	[86]
Sevoflurane	USP7-PTEN axis	Antioxidant	Inhibits USP7, destabilizes PTEN, activates PI3K-AKT signaling, and suppresses ferroptosis	Myocardial ischemia-reperfusion injury	Reduces infarct size, fibrosis inflammation, and myocardial injury	Clinically used inhalational anesthetic for induction and maintenance of general anesthesia	[80]
Lurasidone	SPATA2-CYLD-p53 axis	Antioxidant	Suppresses SPATA2, restores p53 ubiquitination, lowers p53, and increases SLC7A11 and GPX4	Myocardial ischemia-reperfusion injury	Reduces ferroptosis and attenuates myocardial injury	Clinically used atypical antipsychotic for schizophrenia and bipolar depression	[92]
Salvianolic acid B	GPX4 ubiquitin proteasome degradation	Antioxidant	Decreases ubiquitin proteasome degradation of GPX4 and preserves GPX4 expression	Myocardial ischemia-reperfusion injury	Alleviates oxidative stress ferroptosis and myocardial injury	Natural compound from *Salvia miltiorrhiza*; mainly experimental in I/R-ferroptosis studies	[116]
Tangeretin	NAMPT ubiquitination degradation	Antioxidant	Inhibits ubiquitination dependent degradation of NAMPT and stabilizes NAMPT expression	Myocardial ischemia-reperfusion injury	Reduces ferroptosis, alleviates myocardial injury, and improves cell survival	Natural polymethoxyflavone; mainly experimental in I/R-ferroptosis studies	[122]
Peptide from C Phycocyanin	UCHL3	Antioxidant	Upregulates UCHL3 and suppresses ferroptosis associated oxidative injury	Myocardial ischemia-reperfusion injury	Protects myocardium and attenuates ferroptosis	Bioactive peptide derived from C-phycocyanin; experimental agent	[123]
Resveratrol	USP19-Beclin1 axis	Organelle	Regulates USP19-Beclin1 autophagy associated ferroptosis and attenuates oxidative stress	Myocardial ischemia-reperfusion injury	Protects myocardium and attenuates ferroptosis	Natural polyphenol/nutraceutical compound; not an established therapy for ischemic injury	[117]
PRT4165 delivered by targeted nanoparticle system	PRC1-Histone H2A ubiquitination axis	Organelle	Inhibits PRC1, decreases H2AK119 ubiquitination, upregulates Hsp27, promotes glycolysis, and improves mitochondrial function	Myocardial ischemia-reperfusion injury	Suppresses ferroptosis and protects myocardium from ischemia-reperfusion injury	Experimental PRC1 inhibitor delivered by a preclinical nanoparticle system	[108]
Cyanidin 3 glucoside	USP19-Beclin1 axis	Organelle	Suppresses USP19 and promotes K11 linked ubiquitination of Beclin1, reducing ferritinophagy associated ferroptosis	Myocardial ischemia-reperfusion injury	Reduces ferroptosis and alleviates myocardial ischemia-reperfusion injury	Natural anthocyanin compound; mainly experimental in I/R-ferroptosis studies	[105]
ML351	RNF34-PGC1α axis	Organelle	Inhibits 15-HpETE-driven ubiquitin dependent degradation of PGC1α and preserves mitochondrial biogenesis	Myocardial ischemia-reperfusion injury	Inhibits cardiomyocyte ferroptosis, protects injured myocardium, and improves cardiac function	Experimental inhibitor used in mechanistic and preclinical studies	[20]
Honokiol	PINK1-Parkin pathway	Organelle	Reverses PINK1/Parkin silencing	Myocardial ischemia-reperfusion injury	Reduces ferroptosis, oxidative stress, and mitochondrial damage	Natural biphenol compound; widely investigated in I/R injury and ferroptosis-related researches	[118]
Trimetazidine	PINK1-Parkin pathway	Organelle	Promotes mitophagy by increasing PINK1/Parkin pathway activation	Myocardial ischemia-reperfusion injury	Improved cardiac function, reduces mitochondrial damage, myocardial oxidative stress, and infarct size	Synthetic anti-angina cardiovascular drugs; mainly experimental in I/R-ferroptosis studies	[119]
AP39	PINK1-Parkin pathway	Organelle	Activates the PINK1/Parkin pathway	Myocardial ischemia-reperfusion injury	Inhibit mitochondrial autophagy and ferroptosis; improves myocardial fibrosis	Synthetic mitochondria-targeting H_2_S donor; mainly experimental in I/R-ferroptosis studies	[121]
Dipyridamole	RNF126-SLC7A11 axis	Antioxidant	Downregulates RNF126 and reduces ubiquitination mediated proteasomal degradation of SLC7A11	Ischemia-reperfusion injury and doxorubicin induced cardiac injury with additional liver and kidney injury models	Inhibits ferroptosis and protects multiple organs from tissue injury	Clinically used antiplatelet and vasodilatory drug; used as an adjunct to anticoagulation for prevention of thromboembolic complications after cardiac valve replacement	[93]
Nadolol	HOIL-1-IRP2 axis	Iron	Targets HOIL-1-IRP2 signaling, reverses IRP2 and TfR1 upregulation, and restores FPN1	Cerebral ischemia-reperfusion injury	Attenuates brain ferroptosis, reduces infarct injury, and improves neurological status	Clinically used non-selective β-blocker for hypertension and angina	[78]
Tongqiao Huoxue Decoction	ACSL4	Lipid	Promotes ubiquitination and degradation of ACSL4, reduces oxidative stress, and inhibits ferroptosis initiation	Cerebral ischemia-reperfusion injury	Provides neuroprotection and reduces cerebral infarction and injury	Traditional Chinese medicine formula; clinically used in traditional practice but anti-ferroptosis evidence remains preclinical	[115]
Melatonin	MDM2-ACSL4 axis	Lipid	Inhibits ferroptosis through MDM2 mediated ubiquitination of ACSL4	Cerebral ischemia-reperfusion injury	Reduces cerebral ischemic injury brain edema neuronal damage and apoptosis	Endogenous hormone and commonly used sleep-related supplement; anti-ferroptosis use remains experimental	[90]
Melatonin	FBXO10-ACSL4 axis	Lipid	Enhances CYP1B1/20-HETE/PKC signaling induced FBXO10 expression and promotes ACSL4 ubiquitination degradation	Cerebral ischemia-reperfusion injury	Reduces infarct area edema neuronal injury and ferroptosis	Endogenous hormone and commonly used sleep-related supplement; anti-ferroptosis use remains experimental	[87]
Remimazolam	TRIM67-ACSL4 axis	Lipid	Enhances TRIM67 expression and promotes ubiquitination and degradation of ACSL4	Cerebral ischemia-reperfusion injury	Inhibits ferroptosis apoptosis and inflammation and alleviates cerebral injury	Clinically used ultra-short-acting benzodiazepine sedative for procedural sedation	[88]
Rosmarinic acid	KEAP1-NRF2 axis	Antioxidant	Directly binds KEAP1, stabilizes NRF2, and enhances expression of GPX4, SLC7A11, and GCLM	Cerebral ischemia-reperfusion injury	Suppresses neuronal ferroptosis, reduces infarct volume, and improves neurological outcomes	Natural polyphenol; mainly experimental in I/R-ferroptosis studies	[99]
Farudodstat	TRIM56-KLF4 axis	Antioxidant	Acts as a potential TRIM56 inhibitor and prevents KLF4 K48-linked ubiquitination degradation	Cerebral ischemia-reperfusion injury	Reduces neuronal ferroptosis and mitigates cerebral I/R injury	Oral DHODH inhibitor under clinical development; not an approved therapy for ischemic injury	[102]
*Momordica charantia* small extracellular vesicles	TRIM62-GPX4 axis	Antioxidant	Deliver miR-5813b to inhibit TRIM62 and reduce K48 linked ubiquitination of GPX4	Cerebral ischemia-reperfusion injury	Attenuates neuronal ferroptosis and improves neurological recovery	Experimental extracellular vesicle-based intervention	[96]
Piceatannol	USP14-GPX4 axis	Antioxidant	Promotes USP14 mediated deubiquitination of GPX4 and prevents GPX4 degradation	Cerebral ischemia-reperfusion injury	Reduces brain injury, inhibits neuronal ferroptosis, and improves cognitive dysfunction	Natural stilbene compound; mainly experimental in I/R-ferroptosis studies	[19]
Bone marrow mesenchymal stem cell derived exosomal USP10	USP10-SLC7A11 axis	Antioxidant	Stabilizes SLC7A11 by deubiquitination and preserves redox defense	Cerebral ischemia-reperfusion injury	Alleviates neuronal injury and suppresses ferroptosis	Experimental stem cell-derived exosome-based intervention	[76]
Salvianolic acid B	THBS1-OTUD5-GPX4 axis	Antioxidant	Inhibits THBS1-OTUD5 interaction and reduces GPX4 ubiquitination	Cerebral ischemia-reperfusion injury	Reduces endothelial ferroptosis and alleviates brain injury	Natural compound from *Salvia miltiorrhiza*; mainly experimental in I/R-ferroptosis studies	[111]
Salidroside	PINK1-Parkin pathway	Organelle	Upregulates PINK1 and Parkin and activates mitophagy associated ferroptosis control	Cerebral ischemia-reperfusion injury	Reduces ferroptosis, oxidative stress, infarct volume, and neurological injury	Natural phenolic glycoside; mainly experimental in I/R-ferroptosis studies	[120]

Abbreviations: TRIM65, tripartite motif containing protein 65; ALOX5, arachidonate lipoxygenase 5; USP7, ubiquitin-specific protease 7; PTEN, phosphatase and tensin homolog; PI3K, phosphatidylinositol 3-kinase; AKT, protein kinase B; SPATA2, spermatogenesis associated protein 2; CYLD, cylindromatosis; SLC7A11, solute carrier family 7 member 11; GPX4, glutathione peroxidase 4; I/R, ischemia-reperfusion; NAMPT, nicotinamide phosphoribosyltransferase; UCHL3, ubiquitin C-terminal hydrolase L3; USP19, ubiquitin-specific protease 19; PRC1, polycomb repressive complex 1; H2AK119, histone H2A Lys119; Hsp27, heat shock protein 27; K11, Lysine 11; RNF34, ring finger protein 34; PGC1α, peroxisome proliferator activated receptor gamma coactivator 1 alpha; 15-HpETE, 15-hydroperoxyeicosatetraenoic acid; RNF126, ring finger protein 126; IRP2, iron regulatory protein 2; HOIL-1, heme-oxidized IRP2 ubiquitin ligase 1; TfR1, transferrin receptor 1; FPN1, ferroportin 1; ACSL4, acyl-CoA synthetase long-chain family member 4; MDM2, murine double minute 2; FBXO10, F-box only protein 10; CYP1B1, cytochrome P450 family 1 subfamily B member 1; 20-HETE, 20-hydroxyeicosatetraenoic acid; PKC, protein kinase C; TRIM67, tripartite motif containing protein 67; KEAP1, Kelch-like ECH-associated protein 1; NRF2, nuclear factor erythroid 2-related factor 2; GCLM, glutamate-cysteine ligase modifier subunit; TRIM56, tripartite motif containing protein 56; KLF4, Krüppel-like factor 4; K48, Lysine 48; DHODH, dihydroorotate dehydrogenase; TRIM62, tripartite motif containing protein 62; USP14, ubiquitin-specific protease 14; USP10, ubiquitin-specific protease 10; THBS1, thrombospondin 1; OTUD5, ovarian tumour proteases 5; PINK1, PTEN-induced kinase 1; H2S, hydrogen sulfide.

## 6 Conclusion and future directions

Ubiquitin-dependent regulation is closely involved in ferroptosis during I/R injury in the heart and brain [9,78]. Rather than acting only at the final stage of cell death, ubiquitination more often affects earlier steps by regulating iron homeostasis, lipid remodeling, antioxidant defense, and organelle integrity. In this way, it influences whether ischemic cells become susceptible to ferroptosis during reperfusion. Although the heart and brain differ in cell composition, metabolic demand, and local stress responses, both tissues depend on ubiquitin related control of ferroptosis. However, the dominant regulators are not fully the same. In the heart, current studies more often involve lipid remodeling, p53-related antioxidant regulation, and mitochondrial homeostasis. In the brain, more direct regulation of iron transporters, ACSL4, and GPX4 has been described.

A key next step is to identify which ubiquitin events truly drive ferroptosis during early reperfusion. Not all changes observed after ischemia are likely to be causal, as some may reflect secondary responses to established injury. Future studies should therefore distinguish early pathogenic events from later adaptive or reparative changes and define when ferroptosis sensitivity remains reversible. This temporal distinction is important because ubiquitination events that occur in minutes to hours after reperfusion may directly determine early ferroptosis sensitivity, whereas later events occurring over hours to days may reflect sustained inflammation, organelle remodeling, or tissue repair. At present, most studies rely on endpoint measurements and do not fully define when specific E3 ligase- or DUB-dependent events occur. Time-resolved ubiquitinomics, proteomics, and functional rescue studies will be needed to identify which ubiquitin events are early drivers of ferroptosis and which are secondary responses to established injury. This is especially important because the treatment window after reperfusion is short. Another important issue is that the same ubiquitin regulator may act differently across time, tissues, and cell types. Its role may differ between early reperfusion and later remodeling, or between cardiomyocytes, neurons, glial cells, and vascular cells. Parkin is one example, as it appears protective in some settings but harmful in others.

Further progress also requires stronger mechanistic evidence. Many studies report changes in expression or ubiquitination, but fewer define the direct substrate, the ubiquitin linkage type, and the causal relationship with ferroptosis. Therefore, future work should distinguish causal ubiquitination events from secondary or correlative changes after I/R injury. Strong evidence should include substrate-specific binding assays, direct ubiquitination assays, protein stability analysis, linkage-specific validation, ferroptosis-related functional readouts, and rescue experiments using ubiquitination-defective mutants or substrate reconstitution. Time resolved proteomic approaches and spatial analysis in ischemic tissues may help identify the most relevant ubiquitin events during reperfusion. Another challenge is how to translate the findings in animal or cell studies to human disease. Most available evidence comes from cell systems or rodent models, while direct evidence from human ischemic heart and brain tissue remains limited. Studies using patient-derived samples will be important for determining whether the key pathways identified in experimental work are also preserved in clinical ischemic injury.

From a therapeutic perspective, broad inhibition of the ubiquitin proteasome system is unlikely to be the best strategy. A more practical approach is to selectively target disease-related ubiquitin events that act on defined substrates within a useful treatment window. At present, antioxidant related pathways have the strongest support, especially those involving SLC7A11, GPX4, and NRF2. ACSL4 is another repeated target, particularly in cerebral I/R, while iron-related pathways involving IRP2, TfR1, and FPN1 remain less studied but are mechanistically important. Drug repurposing and selective delivery approaches may offer the most realistic near term opportunities.

In summary, ferroptosis in ischemic heart and brain injury is a regulated and potentially modifiable process shaped at multiple upstream levels by ubiquitin dependent signaling. Future progress will depend on identifying early, substrate specific, and tissue relevant ubiquitin events that can still be corrected during reperfusion. A clearer understanding of these events may support more precise treatment strategies for ischemic heart and brain disease.

## Availability of data

Not applicable.

## Consent for publication

This publication is approved by all authors.

## Funding

This work was supported by 10.13039/501100001809National Natural Science Foundation of China (No. 82073849 to Xiu-Ju Luo; No. 82173815 and 81872873 to Jun Peng; and 82360715 to Jing Tian), and 10.13039/501100004735Natural Science Foundation of Hunan Province, China (No. 2021JJ30032 to Xiu-Ju Luo and 2023JJ40514 to Jing Tian).

## CRediT authorship contribution statement

**Yi-Yue Zhang:** Conceptualization, Data curation, Writing – original draft, Writing – review & editing. **Xing-Yu Long:** Data curation, Writing – original draft, Writing – review & editing. **Jing Tian:** Data curation, Funding acquisition, Writing – review & editing. **Xiu-Ju Luo:** Conceptualization, Funding acquisition, Supervision, Writing – review & editing. **Jun Peng:** Conceptualization, Funding acquisition, Supervision, Writing – review & editing.

## Declaration of competing interest

The authors declared no potential conflicts of interest with respect the research, authorship, and/or publication of this article.

## Data Availability

Data will be made available on request.

## Abbreviations

I/R: ischemia-reperfusion
RCD: regulated cell death
ROS: reactive oxygen species
GSH: glutathione
GPX4: glutathione peroxidase 4
DUBs: deubiquitinating enzymes
ACSL4: acyl-CoA synthetase long-chain family member 4
USP14: ubiquitin-specific protease 14
PUFAs: polyunsaturated fatty acids
TFR1: transferrin receptor 1
STEAP3: six-transmembrane epithelial antigen of the prostate 3
NCOA4: nuclear receptor coactivator 4
FPN: ferroportin
AA: arachidonic acid
AdA: adrenic acid
LPCAT3: lysophosphatidylcholine acyltransferase 3
PE: phosphatidylethanolamines
SLC7A11: solute carrier family 7 member 11
FSP1: ferroptosis suppressor protein 1
CoQ10: coenzyme Q10
NADPH: nicotinamide adenine dinucleotide phosphate
ATP: adenosine triphosphate
BBB: blood-brain barrier
UBA1: ubiquitin-like modifier activating enzyme 1
RING: really interesting new gene
HECT: Homologous to the E6-associated protein C-terminus
RBR: RING-between-RING
LUBAC: linear ubiquitin chain assembly complex
IRP2: iron regulatory protein 2
PTEN: phosphatase and tensin homolog
DAMPs: damage-associated molecular patterns
OTUB1: OTU domain-containing ubiquitin aldehyde-binding protein 1
NHLRC1: NHL repeat containing E3 ubiquitin protein ligase 1
HOIL-1: heme-oxidized IRP2 ubiquitin ligase 1
NEDD4L: neural precursor cell expressed developmentally down-regulated 4-like
FTO: fat mass and obesity-associated protein
RNF5: ring finger protein 5
YAP: yes associated protein
TRIM65: tripartite motif containing protein 65
ALOX5: arachidonate lipoxygenase 5
CYP1B1: cytochrome P450 family 1 subfamily B member 1
20-HETE: 20-hydroxyeicosatetraenoic acid
PKC: protein kinase C
FBXO10: F-box only protein 10
MDM2: murine double minute 2
KEAP1: Kelch-like ECH-associated protein 1
NRF2: nuclear factor erythroid 2-related factor 2
CYLD: cylindromatosis
SPATA2: spermatogenesis associated protein 2
HACE1: HECT domain and ankyrin repeat containing E3 ubiquitin protein ligase 1
NAP1L1: nucleosome assembly protein 1-like 1
KLHL8: kelch like protein 8
HSPA5: heat shock protein family member A5
OTUD3: ovarian tumour proteases 3
PLK1: polo-like kinase 1
PI3K: phosphatidylinositol 3-kinase
AKT: protein kinase B
KLF4: Krüppel-like factor 4
PINK1: PTEN-induced kinase 1
PGC1α: peroxisome proliferator activated receptor gamma coactivator 1 alpha
Nur77: nuclear receptor subfamily 4 group A member 1
PRC 1: polycomb repressive complex 1
H2AK119ub: histone H2A Lys119 ubiquitination
SNX5: sorting nexin 5
THBS1: thrombospondin 1
GCLM: glutamate-cysteine ligase modifier subunit
H/R: hypoxia/reoxygenation
MCAO/R: middle cerebral artery occlusion/reperfusion
OGD/R: oxygen-glucose deprivation/reoxygenation
